# Alteration of N^6^-Methyladenosine mRNA Methylation in a Human Stem Cell-Derived Cardiomyocyte Model of Tyrosine Kinase Inhibitor-Induced Cardiotoxicity

**DOI:** 10.3389/fcvm.2022.849175

**Published:** 2022-03-23

**Authors:** Yan Ma, Xian Liu, Yiming Bi, Tianhu Wang, Cheng Chen, Yabin Wang, Dong Han, Feng Cao

**Affiliations:** ^1^National Clinical Research Center for Geriatric Diseases, The Second Medical Center, Chinese People's Liberation Army (PLA) General Hospital, Beijing, China; ^2^Department of Pharmaceutical Sciences, Beijing Institute of Radiation Medicine, Beijing, China

**Keywords:** tyrosine kinase inhibitor (TKI), hiPSC-CMs, N^6^-methyladenosine, FTO, cardiotoxicity

## Abstract

**Background:**

N^6^-methyladenosine (m^6^A) plays important roles in various cardiovascular diseases (CVDs), including cardiac hypertrophy and heart failure. Sunitinib (SUN) is a tyrosine kinase inhibitor (TKI) that is widely used in the treatment of different types of solid and blood tumors, but its efficacy is restricted by a concomitant rise in cardiotoxicities. However, the methylation modification of m^6^A messenger RNA (mRNA) in cardiomyocytes treated with TKI has not been investigated.

**Methods:**

The global m^6^A methylation level of SUN-induced cardiotoxicity was detected by m^6^A dot blot and colorimetric methylation assay. MeRIP-Seq (methylated RNA immunoprecipitation sequencing) and RNA-seq (RNA sequencing, input) were employed to depict the landscapes of transcriptome and epitranscriptome in TKI. Changes in major m^6^A-related enzymes were detected by qRT-PCR and Western blot. In addition, the effects of FTO on SUN-induced cardiotoxicity were evaluated by gain and loss of function studies.

**Results:**

In this study, we observed that the m^6^A methylation level was significantly elevated in SUN-treated human-induced pluripotent stem cell-derived cardiomyocytes (hiPSC-CMs) and paralleled a positively correlated cellular damage level. Through a genome-wide analysis of m^6^A mRNA methylation by methylated RNA immunoprecipitation sequencing (MeRIP-seq) and input RNA sequencing (RNA-seq), we identified a total of 2,614 peaks with significant changes, of which 1,695 peaks were significantly upregulated and 919 peaks were significantly downregulated. Quantitative reverse transcription PCR (RT-qPCR), immunofluorescence, and Western blotting revealed that the RNA demethylase fat mass and obesity-associated protein (FTO) was downregulated, whereas the RNA methylases methyltransferase-like 14 (METTL14) and wilms' tumor 1-associating protein (WTAP) were upregulated. Furthermore, gain- and loss-of-function studies substantiated that FTO is cardioprotective in TKI.

**Conclusion:**

This study deciphered the methylation modification of m^6^A mRNA in hiPSC-CMs post-TKI treatment and determined that FTO may be a promising therapeutic target for TKI-induced cardiotoxicity.

## Introduction

Tyrosine kinase inhibitors (TKIs) have been widely used in the treatment of various types of cancer, some of which are in different stages of clinical development, which shows the importance of tyrosine kinase as the main target of new antitumor drugs (1). However, the widespread use of TKIs was restricted due to their cardiovascular toxicity, which threatened patients' medication compliance and quality of life (2). Therefore, the study of the cardiovascular toxicity mechanism of TKIs is of great significance for circumventing these cardiovascular complications. Sunitinib (SUN), a small-molecule, multitarget receptor tyrosine kinase (RTK) inhibitor, was approved by the US Food and Drug Administration (FDA) in 2006 to treat kidney cancer, gastrointestinal stromal tumors, and endocrine tumors (3). Its targets include vascular endothelial growth factor receptors (VEGFRs), platelet-derived growth factor (PDGFR), and mast/stem cell growth factor receptor (SCFR) (4). In the cardiovascular system, SUN impairs cell signal transduction, cell cycle regulation, and cell metabolism, increasing the incidence of cardiac events in patients with cancer (5). However, cardioprotective strategies based on these mechanisms are controversial and have not been proven in humans, suggesting that SUN-mediated cardiotoxicity may also be mediated by other mechanisms (6).

Ribonucleic acid methylation constitutes more than 60% of all the RNA modifications and N^6^-methyladenosine (m^6^A) is the most prevalent RNA modification in mammalian mRNA and long non-coding RNAs (lncRNAs) (7, 8). The m^6^A modification mainly occurs on adenine in the “RRACH” motif and its state is tightly controlled by “writer” methyltransferases (methyltransferase-like 3 (METTL3), methyltransferase-like 14 (METTL14), and wilms' tumor 1-associating protein (WTAP)), “eraser” demethylases [fat mass and obesity-associated protein (FTO) and Alk B homologue 5 (ALKBH5)], and “reader” m^6^A binding proteins (YT homology domain containing1 (YTHDC1), YTHDC2, YT homology domain family (YTHDF1), YTHDF2, YTHDF3, heterogeneous nuclear ribonucleo protein C (HNRNPC), heterogeneous nuclear ribonucleoprotein A2B1 (HNRNPA2B1), eukaryotic initiation factor 3A (EIF3A), and EIF3C) (9, 10). Ample evidence suggests that m^6^A modification regulates a variety of RNA metabolic processes, such as mRNA stability, splicing, nuclear transport, and translation capabilities (11–13). Given the importance of m^6^A modification in RNA metabolism, we were, thus, curious to discover whether m^6^A modification had potential effects on TKI-induced cardiotoxicity.

Furthermore, previous studies have proposed a cardioprotective role of m^6^A demethylase FTO-mediated demethylating effects in various cardiovascular pathologies. First, reduced FTO expression was observed in failing human hearts and hypoxic cardiomyocytes, thereby increasing m^6^A in RNA and deteriorating cardiomyocyte contractile dysfunction via regulating the methylation of cardiac contractile transcripts (7). Moreover, elevated m^6^A-RNA methylation and FTO repression were causatively involved in myocardial inflammation and dysfunction during endotoxemia in mice (14). Another study showed that FTO overexpression mitigated apoptosis of hypoxia-/reoxygenation-treated myocardial cells by demethylating Mhrt (15). However, the role of FTO in TKI-induced myocardial injury remains to be further revealed.

In this study, we used methylated RNA immunoprecipitation sequencing (MeRIP-seq) and input RNA sequencing (RNA-seq) to study the transcriptome and m^6^A modification epitranscriptome in human-induced pluripotent stem cell-derived cardiomyocytes (hiPSC-CMs) treated with SUN. To gain further insights into the pathological significance of m^6^A modification in TKI-induced cardiotoxicity, the Gene Ontology (GO) and the Kyoto Encyclopedia of Genes and Genomes (KEGG) pathway enrichment analyses were performed on the key genes identified by MeRIP-seq and input RNA-seq. Furthermore, we revealed a cardioprotective role of FTO in SUN-treated hiPSC-CMs. This study is the first study to show that m^6^A methylation may play an indispensable role in TKI-induced cardiotoxicity.

## Materials and Methods

### Cell Culture

Urinary epithelial cell-derived hiPSCs were cultured in Matrigel (Invitrogen, Carlsbad, California, USA)-coated 6-well plates in E8 medium (Invitrogen, Carlsbad, California, USA) containing 0.5% penicillin/streptomycin. HiPSCs were induced to differentiate into cardiomyocytes when cultured at 80% confluence, as previously reported (16, 17). In short, cells were treated with 6 μM of selective inhibition CHIR99021, a selective inhibitor of glycogen synthase kinase 3ß, in roswell park memorial institute (RPMI) medium supplemented with B27 (Invitrogen, Carlsbad, California, USA) for 48 h, followed by 5 μM of Wnt/β-catenin inhibitor (IWR-1), a Wnt antagonist (Sigma-Aldrich), for another 48 h, and the medium was changed every 3 days. On the 10th day, the beating cardiomyocytes were purified by the glucose starvation method for 5 days for further tests.

### Isolation and Culture of Cardiac Microvascular Endothelial Cells (CMECs) and Cardiac Fibroblasts (CFs)

Primary CMECs and CFs were isolated, cultured, characterized, and subjected to subsequent experiments, as previously reported (18, 19).

### Silence and Overexpression of FTO

We employed commercially available ready-to-use lentiviral constructs pLenti-GIII-CMV (Applied Biological Materials Incorporation, CAT. NO 210500610196) and FTO small hairpin RNA (shRNA) lentiviral particles (Santa Cruz Biotechnology Incorporation, CAT. NO sc-75002-V) to overexpress or knockdown FTO in hiPSC-CMs. The transfection process was done as per the manufacturer's instructions. Lentivirus particles were transfected at a multiplicity of infection (MOI) of 20.

### Cell Viability Assay

Cell viability was detected with a Cell Counting Kit-8 (CCK-8) (C0037, Beyotime, Shanghai, China). The culture medium was aspirated and the precoated Matrigel hiPSC-CMs were washed with phosphate-buffered saline (PBS) once. Then, 100 μl of working buffer was added to hiPSC-CMs in 96-well plates and the cells were incubated at 37°C for 30 min in the dark. A microplate reader (Tecan, Switzerland) was used to automatically measure the absorbance at a wavelength of 450 nm.

### Drug Treatment

Our preliminary results showed that the half-maximal inhibitory concentration (IC50) of SUN treatment for 24 h (cell viability serving as the readout) is around 6 μM (Supplementary Figure S1A); thus, the subsequent experiments were carried out with 6 μM SUN (SU11248, Sellect, Shanghai, China) treatment for 24 h and equal volume of dimethyl sulfoxide (DMSO) treatment for 24 h served as the control group. For FTO Demethylase inhibitor (FB23-2) (S8837, Sellect, Shanghai, China) treatment, 20 μM FB23-2 was added simultaneously with SUN for 24 h. The concentration of FB23-2 was chosen based on a previous report (20).

### Western Blot Analysis

Cell protein was extracted with Radio-Immunoprecipitation Assay (RIPA) Lysis Solution (P0013C, Beyotime, Shanghai, China) from hiPSC-CMs for Western blot detection. Protein extractions and molecular weight standards were separated by 10% sodium dodecyl sulfate-polyacrylamide gel electrophoresis (SDS-PAGE) gels and transferred to poly vinyli dene fluoride (PVDF) membranes (Bio-Rad, USA). After blocking, the membrane was incubated with primary antibodies [methyltransferase-like 3, (METTL3) ab195352, Abcam; METTL14, ab220030, Abcam; FTO, ab126605, Abcam; ALKBH5 aa302-330, LifeSpan Biosciences; WTAP, 56501, Cell Signaling Technology; and glyceraldehyde-3-phosphate dehydrogenase (GAPDH) ab8245, Abcam] at 4°C overnight in a 5% bovine serum albumin (BSA) blocking solution. After being washed with Tris-Buffered Saline and Tween (TBST) buffer, the membrane was incubated in 5% blocking buffer for 1 h at room temperature with the secondary antibody (1:800 dilution, 7074, Cell Signaling Technology, USA) at the recommended dilution. Protein bands were detected with the enhanced chemiluminescence (ECL) chemiluminescent kit (P0018S, Beyotime, Shanghai, China) in a dark room and assessed with Image Lab software (Bio-Rad, USA).

### N^6^-Methyladenosine Dot Blot

The mRNA was isolated with the Dynabeads® mRNA Purification Kit (61006, Invitrogen, Carlsbad, California, USA) and the purity of mRNA was detected by the NanoDrop method for further tests. The serially diluted mRNA was denatured at a high temperature of 95°C and cooled immediately after denaturation. The 2 μl sample was transferred directly onto a nucleic acid-optimized nylon membrane (1620153, Bio-Rad, USA). After a regimen UV cross-linking and methylene blue (M4591, Sigma-Aldrich, USA) staining, the membrane was blocked by soaking in 5% BSA buffer and incubated with the anti-m^6^A antibody (ab284130, Abcam, Shanghai, China) in 5% BSA for 30 min at room temperature. Then, the membrane was incubated with horseradish peroxidase (HRP)-conjugated secondary antibody (ab97051, Abcam, Shanghai, China) for 30 min, followed by incubation with the ECL reagent (P0018S, Beyotime, Shanghai, China) for 1 min, covered with plastic wrap, and exposed to different lengths of exposure in a dark room. The test sample was compared with the signal of the standard sample to detect its concentration.

### m^6^A RNA Methylation Assay (Colorimetric)

The m^6^A RNA Methylation Assay Kit (ab185912, Abcam, Shanghai, China) was used to measure the m^6^A level of mRNA. According to the instructions, 80 μl of binding solution was added to each well and negative control, diluted positive control, and 200 ng of mRNA were added to each well and incubated at 37°C for 90 min. Fifty microliter of diluted capture antibody was added for incubation at room temperature for 60 min and then 50 μl of diluted detection antibody was added to each well for 30 min. Finally, 100 μl of developing solution was used for the reaction and after incubation in the dark at room temperature for 10 min, stop solution was added and the absorbance was measured at 450 nm.

### Lactate Dehydrogenase (LDH) Release

The LDH Release Detection Kit (C0016, Beyotime, Shanghai, China) was used to detect cell cytotoxicity according to the instructions. Sixty microliter of LDH detection working solution was added to each well. The sample was incubated at room temperature (~25°C) in the dark for 30 min. Then, the absorbance was measured at 490 nm.

### Quantitative Reverse Transcription PCR (RT-qPCR)

Total RNA was extracted from hiPSC-CMs using the RNAsimple Total RNA Kit (DP419, Tiangen, Beijing, China). One microgram of total RNA was used for complementary DNA (cDNA) synthesis reaction, as previously described (21). The isolated mRNA was reverse transcribed into cDNA with the High Capacity cDNA Reverse Transcription Kit (4368814, Invitrogen, Carlsbad, California, USA) and cDNA was amplified with the Takara's Perfect Real-Time PCR Kit (RR037A, Takara Bio, Otsu, Japan). The primers and probes were ordered from TaqMan (Invitrogen, Carlsbad, California, USA). The relative level of each mRNA was quantified by GAPDH and expressed as a relative ratio.

### Methylated RNA Immunoprecipitation Sequencing

Guangzhou Epibiotek Corporation Ltd. (Guangzhou, China) provided the MeRIP-seq service. Briefly, m^6^A RNA immunoprecipitation was performed with the GenSeq™ m^6^A RNA IP Kit (GE-ET-001, GenSeq Incorporation, China) according to the manufacturer's instructions. Both the input samples were obtained by ribosomal RNA (rRNA) removal and smart principles and first-strand cDNA PCR-enriched library fragments were synthesized. The magnetic bead library fragments were purified by DNA and the ultrafine RNA methylated m^6^A detection library was obtained. The library quality was evaluated using the Bioptic Qsep100 Analyzer (Agilent Technologies Incorporation, USA). Library sequencing was performed on an Illumina HiSeq instrument in PE150 sequencing mode.

### Methylated RNA Immunoprecipitation Sequencing Data Processing

Cutadapt (version 2.5) was used to trim adapters and filter for sequences. The remaining reads were then aligned to the human Ensemble genome GRCh38 (mouse Ensemble genome GRCm38) using Hisat2 aligner (version 2.1.0) under the following parameters: “–rna-strandness RF.” m^6^A peaks were identified using the exome Peak R package (version 2.13.2) under the parameter: “peak_cutoff_*p*-value = 0.05, peak_cutoff_false discovery rate (FDR) = NA, and fragment_length = 200.”

Differential m^6^A peaks were identified using the exome Peak R package under the following parameters: “peak_cutoff_*p*-value = 0.05, peak_cutoff_FDR = NA, and fragment_length = 200.” The GO and the KEGG analyses were performed using the cluster profile R package (version 3.6.0). m^6^A RNA-related genomic features were visualized using the Guitar R package (version 1.16.0). Identified m^6^A peaks with p-values <0.05 were chosen for the *de-novo* motif analysis using homer (version 4.10.4) under the parameter “-len 6-rna.”

### Long RNA-seq

The Epi™ Mini LongRNA-SEQ Kit (E1802, Epibiotek, Guangzhou, China) and the Epi™ DNA Clean Beads Kit (R1809, Epibiotek, Guangzhou, China) was used for long RNA sequencing. DNase I was added to the RNA samples and digested at 37°C for 30 min to remove the residual DNA in the samples and the RNA was purified and recovered by magnetic beads. rRNA removal and RNA fragmentation: 5XRT buffer was added to sample RNA, a rRNA probe, and a temperature gradient reaction was used to fragment RNA samples and remove rRNA. Synthesis of first-strand cDNA: EpiScript™ IV, RNase inhibitor, DL-Dithiothreitol (DTT), Template-Switching oligonucleotide (TSO), and random primers were added to the RNA samples in Step 2. After mixing with the wall of the tube, rapid centrifugation was carried out in the PCR machine according to the following procedures: 37°C, 90 min; 70°C, 15 min. 2XpfuMax HiFi PCR ProMix and sequencing primers were added to the first-strand cDNA samples and then amplified in a PCR apparatus after mixing. The Epi™ DNA Clean Beads were used to purify PCR products in a 1X ratio. DNA fragments (300–400 bp) were recovered from the purified products with magnetic beads in a 0.65/0.2X ratio for a second round of PCR amplification to enrich 300–400 bp DNA fragments. The Bioptic Qsep100 Analyzer was used to conduct quality inspection of the library to detect whether the size distribution of the library conformed to the theoretical size.

### Ribonucleic Acid Sequencing Data Processing

Cutadapt (version 2.5) was used to trim adapters and filter for sequences and the remaining reads were then aligned to the human Ensemble genome GRCh38 (mouse Ensemble genome GRCm38) using Hisat2 aligner (version 2.1.0) under the parameter “–rna-strandness RF.” The reads mapping the genome were calculated using feature counts (version 1.6.3). Differential gene expression analysis was performed using the DESeq2 R package. Enrichment analysis was performed using the clusterProfiler R package for the GO terms and the KEGG database pathways.

### Immunostaining and Immunofluorescence Analysis

Human-induced pluripotent stem cell-derived cardiomyocytes were separated and placed in 6-well plates (Corning, New York, USA). The combined staining of α-actinin (ab137346, Abcam), immunoglobulin G (IgG) H&L (Alexa Fluor® 488) (ab150077, Abcam), and propidium iodide (PI) 1 μg/ml (ST511, Beyotime, Shanghai, China) was used to detect cardiomyocyte death. The nucleus was stained with 4′,6-diamidino-2-phenylindole (DAPI) (C1002, Beyotime, Shanghai China) and the dead cells were labeled with PI to pass through the damaged cell membrane. A Nikon A1R HD25 confocal microscope was used to capture images. The total number of cells in the PI-positive and five randomly selected fields was counted using ImageJ software by a researcher blinded to the treatment assignments. A manual pipeline (CellProfiler, Broad Institute of Massachusetts Institute of Technology (MIT) and Harvard in Cambridge) was used to determine the cell surface area (22). Briefly, 5 to 6 random pictures were taken with 20X magnification and 150–200 cells/per condition were analyzed in order to determine cell size following the instructions of the software.

### Statistical Analyses

Continuous data are expressed as the mean ± SD unless otherwise specified. Comparisons between the two or more groups were performed using the Student's *t*-test and ANOVA for normal variables or the Mann–Whitney *U* test and the Kruskal–Wallis test for non-normal variables. R software (version 3.4.2) and GraphPad Prism software (version 8.00) were used for statistical analysis. Biological replicates (individual mice) are shown as individual data points superimposed on bar charts. Significance was conventionally accepted at *p* < 0.05.

## Results

### Global m^6^A Level Was Upregulated in SUN-Injured hiPSC-CMs

The treatment dose and duration of SUN were determined based on preliminary experiments. Our preliminary results showed that the IC50 of SUN treatment for 24 h (cell viability serving as the readout) is around 6 μM (Supplementary Figure S1A); thus, the subsequent experiments were carried out with 6 μM SUN treatment for 24 h and equal volume of DMSO treatment for 24 h served as the control group. Furthermore, we also explored the time kinetics of 6 μM SUN in hiPSC-CMs; these results were given in Supplementary Figure S1B.

**Figure 1:**
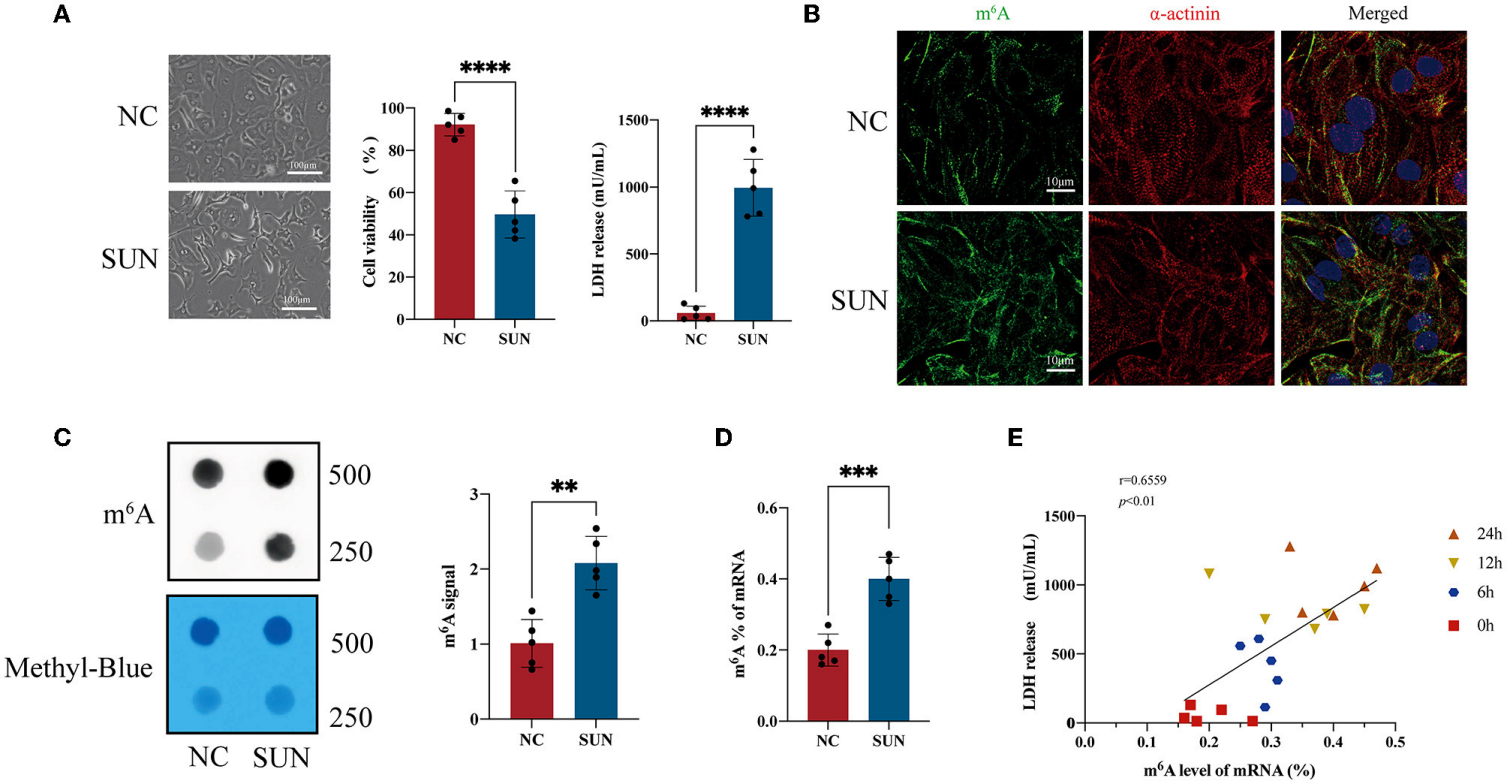
The global N^6^-methyladenosine (m^6^A) methylation level of sunitinib (SUN)-treated human-induced pluripotent stem cell-derived cardiomyocytes (hiPSC-CMs). **(A)** Representative images after SUN treatment for 24 h and measurement of lactate dehydrogenase (LDH) release level and cell viability are shown (*n* = 5). **(B)** The levels of m^6^A methylation in the normal control (NC) and SUN groups detected by m^6^A antibody-based immunofluorescence. Red denotes α-actinin, green denotes m^6^A, and blue denotes 4′,6-diamidino-2-phenylindole (DAPI). **(C)** The m^6^A level of total RNA of hiPSC-CMs is indicated by an m^6^A dot blot. Corresponding RNAs were loaded equally by a 2-fold serial dilution with 500 ng and 250 ng of methylene blue staining served as a loading control. **(D)** The RNA m^6^A level detected by the colorimetric method. **(E)** The m^6^A level of messenger RNA (mRNA) and the level of LDH release exhibit a time-dependent gradual increase after SUN treatment and the two indicators are positively correlated. “**” indicates *p* < 0.01, “***” indicates *p* < 0.001, and “****” indicates *p* < 0.0001.

Figure 1A shows the optical microscope morphology of hiPSC-CMs treated with 6 μM of SUN for 24 h. The cell surface area decreased, accompanied by a significant elevation in LDH release and a significant reduction in cell viability in SUN-treated hiPSC-CMs. Furthermore, the immunofluorescence results suggested that the global m^6^A levels in the SUN group increased and that the structure of myocardial sarcomeres became disorganized (Figure 1B). The m^6^A dot blot validated that the global m^6^A level was indeed elevated in the SUN group (Figure 1C). In addition, the colorimetric kit method also verified the upregulation of the global m^6^A level (Figure 1D). Linear regression was used to analyze the relationship between the mRNA m^6^A level and LDH release of hiPSC-CMs after SUN treatment and it was found that a positive correlation was identified between the global m^6^A level and LDH release (*r* = 0.6096, *p* < 0.01) and the global m^6^A level and LDH release gradually increased as the treatment time was prolonged (Figure 1E). Overall, these results indicated that the dysregulated m^6^A modification in SUN-injured hiPSC-CMs may play important roles in TKI-induced cardiotoxicity.

### Overview of the m^6^A Methylation Map in SUN-Injured hiPSC-CMs

**Figure 2:**
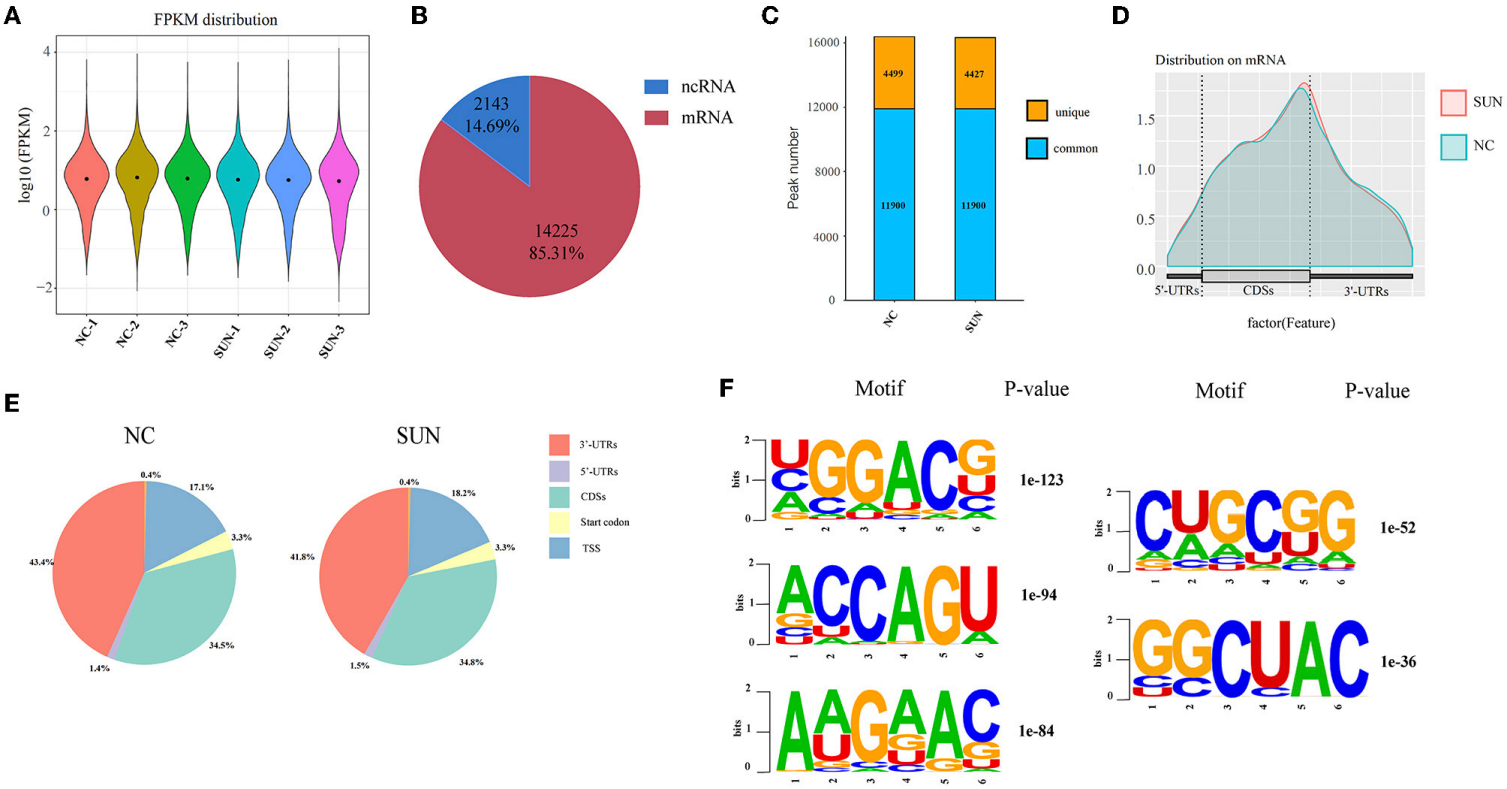
Overview of the m^6^A methylation map in SUN-treated hiPSC-CMs. **(A)** Sequencing sequences were compared to the genome and the distribution intensity and abundance of normalized expression quantities are described. **(B)** Pie chart showing the distribution of m^6^A peaks in mRNAs and non-coding RNAs (ncRNAs). **(C)** The histogram displays the unique and common m^6^A peaks in the two sets of mRNAs. **(D)** The density curve shows the distribution of the m^6^A peak on the transcript, which is divided into three parts: 5′-UTR, CDSs, and 3′-UTRs. **(E)** Pie charts showing the proportion of the m^6^A peak distribution in the NC and SUN groups. **(F)** The top five motifs enriched across the m^6^A peaks. SUN, sunitinib; CDS, coding DNA sequence; UTR, untranslated region.

Next, to further decipher the role of elevated m^6^A in SUN-injured hiPSC-CMs, three biological copies of hiPSC-CMs from either the normal control (NC) group or the SUN group were sent for MeRIP-seq and m^6^A MeRIP enrichment regions (peaks) were analyzed after sample normalization (Figure 2A). The m^6^A modification mostly occurred in mRNAs (Figure 2B). A total of 16,399 m^6^A peaks from 4,499 coding gene transcripts (mRNAs) were identified in the NC group. In the SUN group, there were 16,732 m^6^A peaks within 4,427 mRNAs (Figure 2C). To reveal the preferential distribution of m^6^A in transcripts, the metagene profiles of all the identified m^6^A peaks in the entire transcriptome were probed. The results show that the m^6^A peak is preferentially enriched in two sets of coding DNA sequences (CDSs) and the 3′-untranslated region (UTR) (Figures 2D,E). To learn whether a consensus motif existed in the identified m^6^A peaks, we used HOMER software to map the m^6^A methylation. The results showed that m^6^A mainly exists in the consensus sequence of 5-RRAH-3′ and 5-RRAH-3′ (R = A or G; H = A, C, or U) (14). Among the identified m^6^A peaks, the top five conserved motifs are shown in Figure 2F, which was consistent with the well-known “RRACH” consensus motif of m^6^A modification.

### Conjoint Analysis of the MeRIP-seq and RNA-seq Data in SUN-Injured hiPSC-CMs

**Figure 3:**
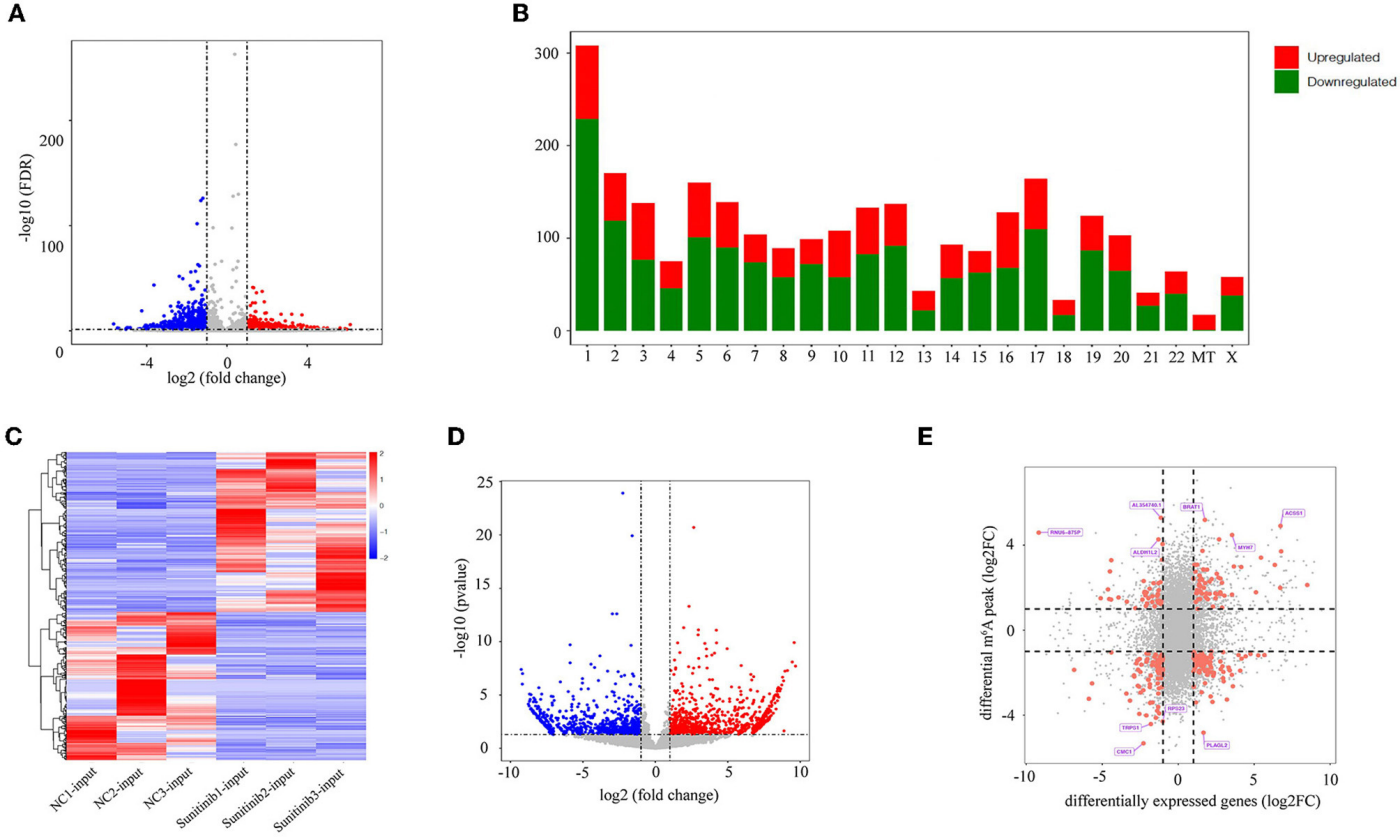
Joint analysis of methylated RNA immunoprecipitation sequencing (MeRIP-seq) and RNA sequencing (RNA-seq) data. **(A)** The volcano plot shows the difference in m^6^A methylation peaks, fold changes ≥2, and *p*-values < 0.05. The red image represents the high methylation peak of m^6^A and the blue image represents the low methylation peak of m^6^A. **(B)** The histogram shows the distribution of the m^6^A peak on chromosomes. **(C)** Heatmap showing upregulated and downregulated mRNAs. **(D)** The volcano map shows differentially expressed genes, *p*-value < 0.05, and double change ≥2. The red area represents upregulated genes and the blue area represents downregulated genes. **(E)** Four-quadrant diagram showing the relationship between mRNA m^6^A methylation and mRNA expression.

To further clarify the changes in m^6^A methylation associated with TKI-induced cardiotoxicity, we performed a conjoint analysis of the MeRIP-seq and RNA-seq data. As shown in Figure 3A, 20,072 m^6^A peaks (representing 9,892 genes) were identified and there were 2,614 differentially methylated peaks (representing 2,066 genes), among which 919 differentially methylated peaks had hypermethylation and 1,695 differentially methylated peaks had hypomethylation at the log fold change cutoff of (±1) and FDR cutoff of <0.05. The top 10 hypermethylated genes and the top 10 hypomethylated genes are shown in Table 1. The analysis of the differentially methylated peak (DMPeaks) distribution at different chromosome loci revealed that the chromosomes with the most m^6^A methylation were chromosome 1 with 308 m^6^A methylation peaks, chromosome 2 with 170 m^6^A methylation peaks, and chromosome 17 with 164 m^6^A methylation peaks (Figure 3B). In parallel, RNA-seq was used to determine the transcriptome profile of altered genes. We identified 1,906 differentially expressed genes (DEGs) between the NC and SUN groups, including 990 upregulated DEGs and 916 downregulated DEGs (fold changes 2, *p* < 0.05; Figures 3C,D). The top 10 upregulated mRNAs and the top 10 downregulated mRNAs are shown in Table 2. Furthermore, among the 20,072 m^6^A peaks (representing 9,892 genes) identified, there were 2,614 differentially methylated peaks (representing 2,066 genes), 919 hypermethylated peaks, and 1,695 hypomethylated peaks. Accordingly, we identified 244 mRNAs with significant changes in their m^6^A peaks and levels and they could be divided into four quadrants: both the mRNA expression and m^6^A peaks were upregulated (55), mRNA and m^6^A peaks were both downregulated (74), m^6^A peaks were upregulated and mRNA peaks were downregulated (37), and m^6^A peaks were downregulated and mRNA peaks were upregulated (78) (Figure 3E). We have validated the transcriptomic study by examining the gene expression level of top 5 upregulated and top 5 downregulated protein coding genes among the 244 intersection genes by RT-qPCR assay. The RT-qPCR results were mostly consistent with RNA-seq (Supplementary Figure S2), which might be helpful to solidify our sequencing result. The list of 244 DEGs with significant differential m^6^A peaks is given in Supplementary Table S2. Their GO term for enrichment analysis is given in Supplementary Figure S3. The GO analysis showed that the biological functions of the 244 mRNAs were mainly enriched in mitogen-activated protein kinase (MAPK) and p53 signaling pathway, while the KEGG analysis repetitiously pointed to apoptotic signaling pathways.

**Table 1:** List of the top 10 hypomethylated genes and the top 10 hypermethylated genes.

**Genes**	**Description**	**Chromosome**	**Peak start**	**Peak end**	**log_**2**_ Fold change**	***P*-value**	**Up/down**
FAM69B	Family with sequence similarity 69 member B	9	136,723,693	136,724,020	−5.65	1.023e-8	Down
ATXN1	Ataxin 1	6	16,300,520	16,300,791	−5.46	1.58e-4	Down
SLC30A6	Solute carrier family 30 member 6	2	32,224,109	32,224,289	−5.39	4.07e-4	Down
JAM2	Junctional adhesion molecule 2	21	25,716,872	25,717,262	−5.04	0.0036	Down
NRDE2	Nuclear RNAi defective 2	14	90,270,343	90,270,584	−5.02	3.31e-4	Down
MANEL	Mannosidase like protein	1	37,799,732	37,800,181	−4.98	5.89e-5	Down
PLAGL2	Pleiomorphicad-enomagene like 2	20	32,195,399	32,195,639	−4.82	7.41e-5	Down
TRPS1	Trichorhinophalangeal syndrome type 1	8	115,412,594	115,412,805	−4.42	0.0024	Down
TSPAN5	Tetraspanin 5	4	98,472,253	98,472,524	−4.33	0.0013	Down
RPS23	Ribosomal protein S23	5	82,275,563	82,275,772	−4.29	0.0043	Down
RGMB	Repulsive guidance molecules B	5	98,795,779	98,796,079	6.15	4.47e-8	Up
TTN	Titin	2	178,693,982	178,702,575	5.98	0.0035	Up
NEBL	Nebulette	10	20,831,577	20,845,420	5.89	0.0010	Up
FABP3	Fat acid binding protein 3	1	31,369,427	31,372,984	5.64	1.70e-4	Up
STOX2	Stork head box 2	4	184,009,557	184,009,768	4.98	0.0051	Up
DYRK3	Dual-specificity tyrosine-(Y)-phosphorylation regulated kinase 3	1	206,647,569	206,647,839	4.94	0.0054	Up
BRAT1	Breast cancer type 1 associated ring domain 1	7	2,542,069	2,542,339	4.83	1.15e-4	Up
ZNF697	Zinc finger protein 697	1	119,624,014	119,625,981	4.52	1.82e-5	Up
LSM14A	RNA-associated protein 55A,	19	34,228,887	34,229,157	4.49	1.70e-5	Up
CCDC184	Coiled-coil domain containing 184	12	48,184,769	48,185,096	4.44	4.57e-4	Up

**Table 2:** List of the top 10 upregulated messenger RNAs (mRNAs) and the top 10 downregulated mRNAs.

	**Description**	**log2 Fold change**	***P*-value**	**Up/down**
CATSPER2	The cation channel of sperm receptor 2	−9.1720856	1.01E-07	Down
ITGB7	Integrin Beta 7	−8.6837258	3.99E-05	Down
CLDN20	Claudin 20	−8.6824254	8.49E-06	Down
GMPR	Guanosine monophosphate reductase	−8.6137908	0.00021296	Down
USP50	Ubiquitin-specific peptidase 50	−8.5858481	9.24E-06	Down
PCDHA7	Protocadherin alpha 7	−8.402541	0.00040126	Down
ZP1	Zona pellucida glycoprotein 1	−8.3886407	0.00054659	Down
SLC37A4	Glucose-6-phosphate transporter member 4	−8.3131318	2.82E-05	Down
KCNC1	Potassium voltage-gated channel 1	−8.2366521	0.00065662	Down
LPAR4	Lysophosphatidic acid receptor 4	−8.1447607	0.00105897	Down
PGF	Vascular endothelial growth factor	9.66737242	2.05E-08	Up
AVPI1	Arginine vasopressin-induced 1	9.44284665	8.06E-09	Up
ANKRD45	Ankyrin repeat domain 45	9.11522806	5.18E-08	Up
TNFRSF10A	Tumor necrosis factor receptor superfamily member 10A	8.94685039	5.80E-08	Up
MYOCD	Myocardin	8.87253755	0.02317118	Up
NPTX1	Neuronal pentraxin 1	8.84367054	4.82E-07	Up
SLCO4A1	Solute carrier organic anion transporter family, member 4A1	8.77762835	2.14E-07	Up
MEDAG	Mesenteric estrogen-dependent adipogenesis protein	8.60841757	2.70E-06	Up
IGSF9	Immunoglobulin superfamily, member 9	8.51141356	1.63E-05	Up
SULT1C2	Sulfotransferase family, cytosolic 2C	8.49784929	2.13E-06	Up

### The GO and the KEGG Enrichment Analyses Revealed the Biological Information Underlying DEGs and Differentially Methylated Genes (DMGs)

**Figure 4:**
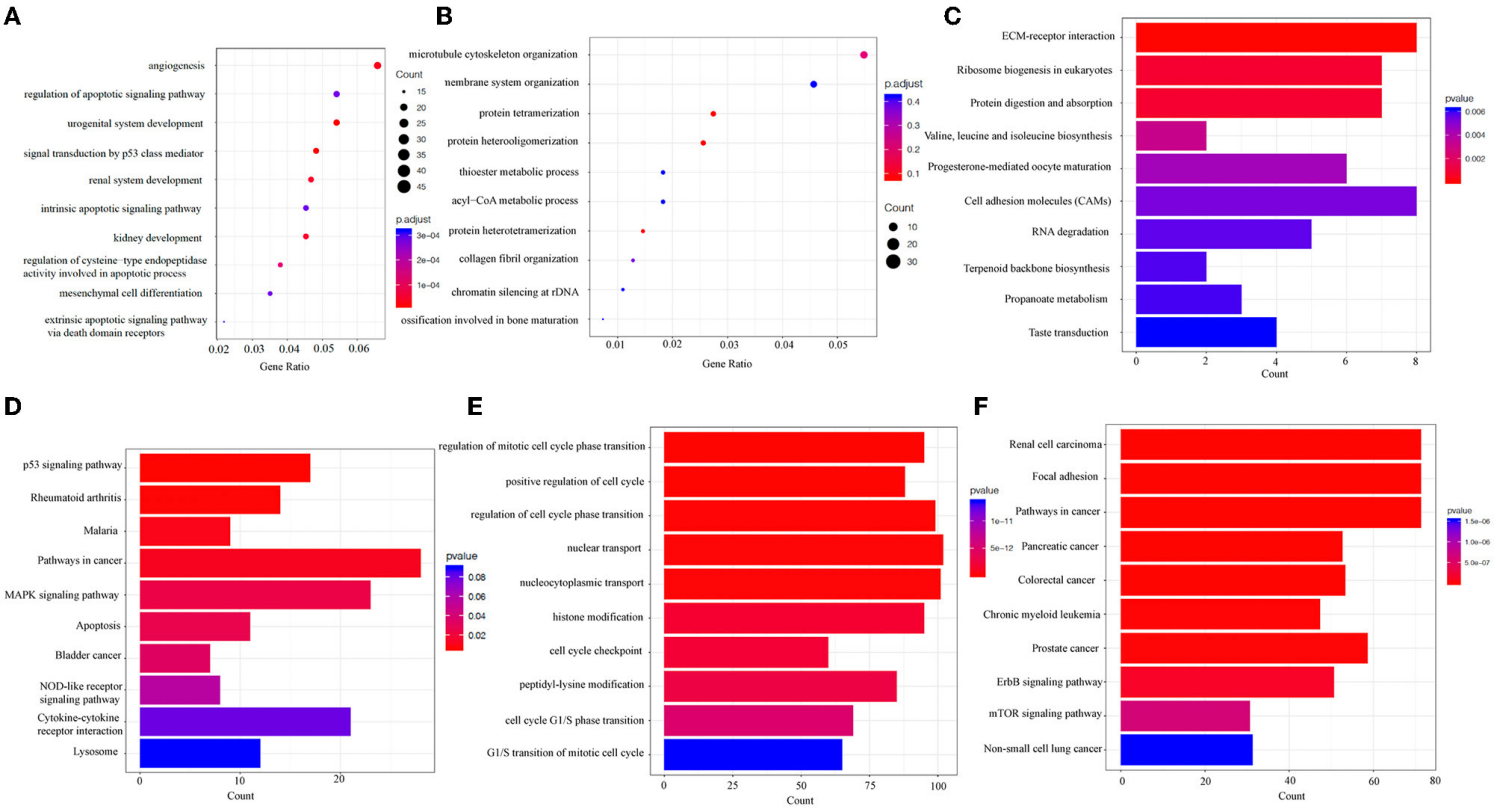
The Gene Ontology (GO) and the Kyoto Encyclopedia of Genes and Genomes (KEGG) analyses reveal the biological information behind the differences in mRNA expression levels and m^6^A methylation modification. **(A,B)** The top 10 enriched GO terms of upregulated and downregulated DEGs. **(C,D)** The top 10 enriched KEGG pathways of upregulated and downregulated DEGs. **(E,F)** The top 10 enriched GO terms and KEGG pathways of DMGs. DEGs, differentially expressed genes. DMGs, differentially methylated genes. “Gene ratio,” number of genes annotated to the specific GO term/number of all the genes with the GO database annotations. “Count,” number of genes annotated to the specific GO term.

To explore the physiological and pathological significance of the DEGs and DMGs, the GO and the KEGG pathway analyses were performed on the key genes identified. The GO analysis showed that the biological functions of upregulated DEGs were mainly enriched in the regulation of angiogenesis and apoptosis signaling pathways (Figure 4A). The downregulated DEGs were mainly involved in microtubule cytoskeleton organization, endomembrane system organization, protein tetramerization, and protein heterodimerization (Figure 4B). Through the KEGG analysis, the upregulated DEGs were mainly enriched in pathways in cell adhesion molecules (CAMs), extracellular matrix (ECM)-receptor interaction, and ribosome biogenesis in eukaryotes (Figure 4C). The downregulated DEGs were mainly involved in cancer pathways, the MAPK signaling pathway, cytokine–cytokine receptor interactions, and the p53 signaling pathway (Figure 4D). In addition, we also performed enrichment analyses on DMGs. These genes were mainly enriched in pathways such as nuclear transport, nucleocytoplasmic transport, regulation of cell cycle phase transition, and histone modification (Figure 4E). The KEGG analyses showed that these DMGs were related to pathways in cancer, focal adhesion, regulation of actin cytoskeleton, and protein processing in the endoplasmic reticulum (Figure 4F).

### Expression of FTO Was Downregulated, While the Expressions of MELLT14 and ALKBH5 Were Upregulated in SUN-Treated hiPSC-CMs

**Figure 5:**
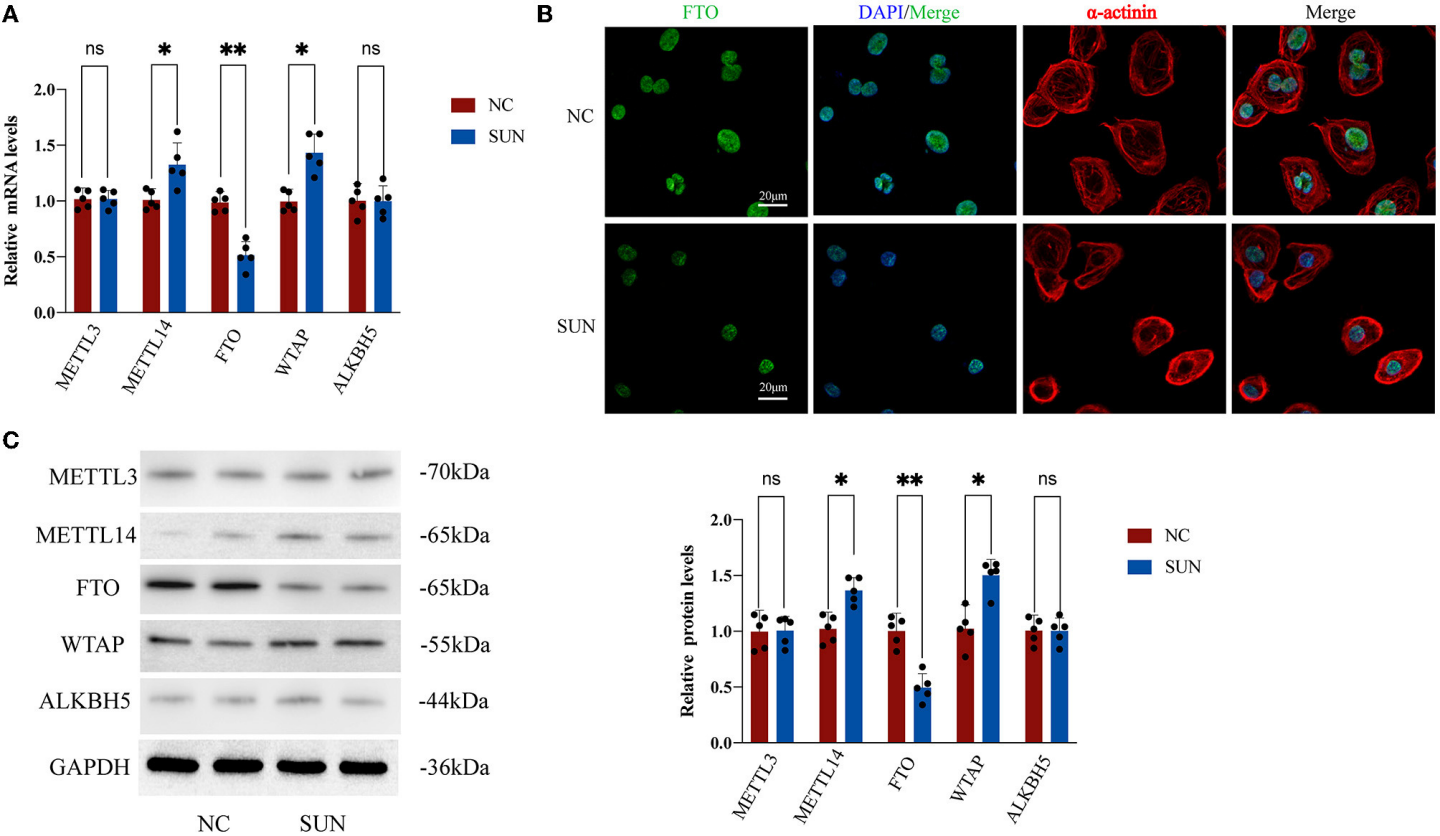
Changes in m^6^A methyltransferase and demethylase expression levels in hiPSC-CMs treated with SUN. **(A)** Reverse transcription PCR (RT-PCR) of METTL3, ALKBH5, fat mass and obesity-associated protein (FTO), METTL14, and WTAP; the NC group (*n* = 5) and the SUN group (*n* = 5). **(B)** The levels of FTO expression in the NC and SUN groups as detected by immunofluorescence. Red denotes α-actinin, green denotes FTO, and blue denotes DAPI. **(C)** The protein expression levels of methyltransferase and demethylase as detected by Western blot (*n* = 5). NS, non-significant, “*” indicates *p* < 0.05 and “**” indicates *p* < 0.01.

To further explore whether the m^6^A modification enzyme was involved in SUN-induced hiPSC-CM injury, we examined the expression levels of major methyltransferases and demethylases. Compared with the NC group, the mRNA expression levels of the methylases WTAP and METTL14 in the SUN group were significantly upregulated (*p* < 0.05), whereas the mRNA expression level of FTO (demethylase) was significantly downregulated in the SUN group (*p* < 0.01) (Figure 5A). The downregulation of FTO was again verified in the SUN group by immunofluorescence (IF) staining (Figure 5B). However, the expression of the other two enzymes, METTL3 and ALKBH5, was not altered in SUN-treated hiPSC-CMs. Consistent with the mRNA expression data, the protein levels of WTAP, METTL14, and FTO exhibited similar trends in SUN-treated hiPSC-CMs (Figure 5C). Collectively, these results indicated that the downregulated FTO as well as the upregulated METTL14 and WTAP might account for the increased global m^6^A level in SUN-injured hiPSC-CMs.

### FTO Downregulation Aggravated SUN-Induced hiPSC-CM Injury

**Figure 6:**
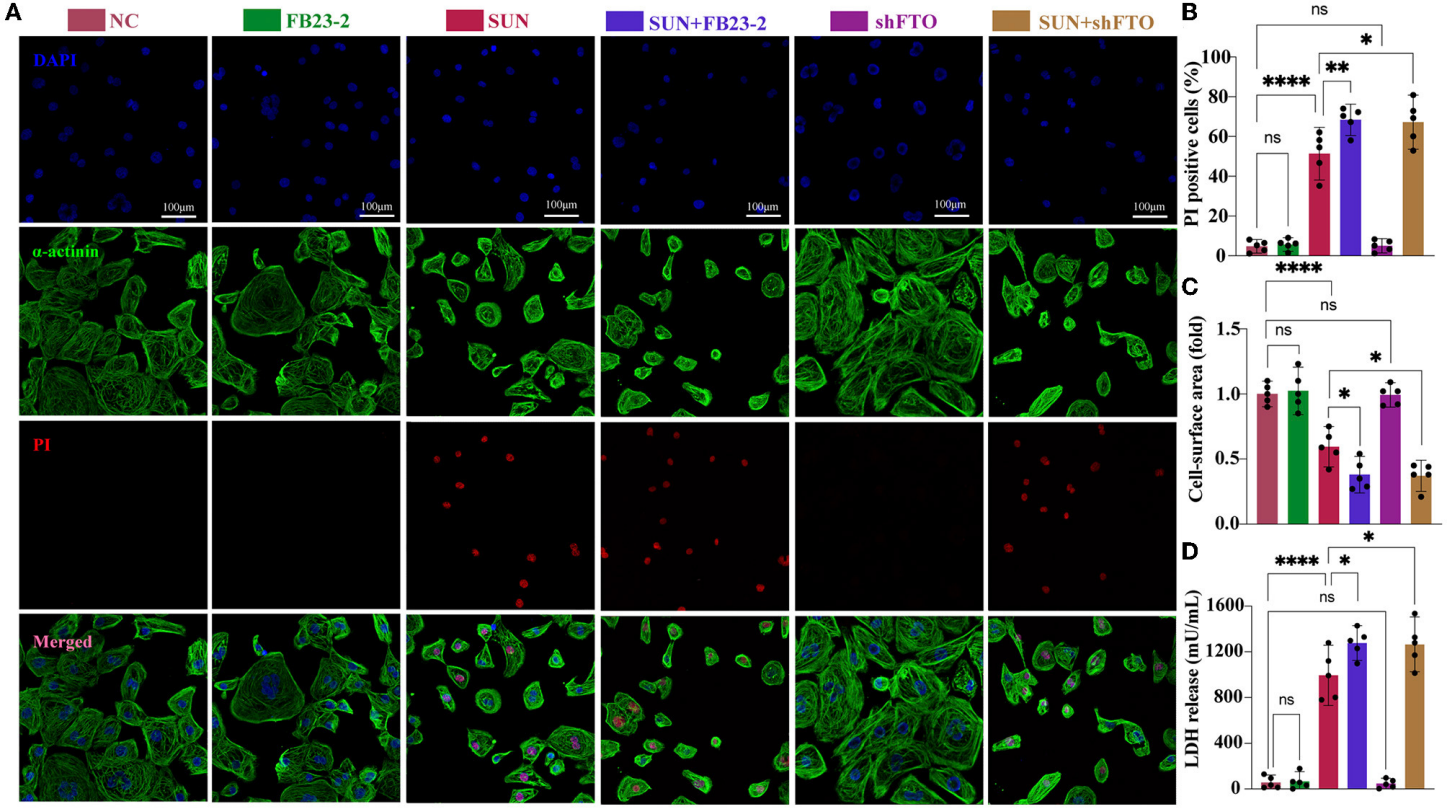
The FTO inhibitor FB23-2 and knocking down of FTO aggravated SUN-induced hiPSC-CM injury. **(A)** Multiple immunofluorescences staining of DAPI (blue), propidium iodide (PI) (red), and α-actinin (green) to detect hiPSC-CM death. **(B)** The proportions of PI-positive cardiomyocytes (*n* = 5). **(C)** The cell surface area was detected and analyzed by CellProfiler pipeline (*n* = 5). **(D)** Measurement of LDH release levels (*n* = 5). NS, non-significant, “*” indicates *p* < 0.05, “**” indicates *p* < 0.01, and “****” indicates *p* < 0.0001.

Next, we employed FB23-2, a potent and selective FTO inhibitor that directly binds to FTO and selectively inhibits FTOs m^6^A demethylase activity to examine the role of FTO in SUN-induced hiPSC-CM injury. For FB23-2 treatment, 20 μM FB23-2 was added simultaneously with SUN for 24 h. The concentration of FB23-2 was chosen based on a previous report (20). We examined the effect of FB23-2 on global m^6^A level by m^6^A dot blot. The results verified a ~1.7-fold upregulation of global m^6^A level. Cell death was evaluated by PI staining after 24 h of treatment. Interestingly, SUN-induced hiPSC-CM death was exacerbated in the FB23-2 + SUN group (Figures 6A,B). This effect was not observed in normal hiPSC-CMs, indicating that FB23-2 alone would not affect the cell viability of hiPSC-CMs. Furthermore, SUN-induced hiPSC-CM atrophy was also aggravated in the FB23-2 + SUN group, as evidenced by a smaller cell surface area (Figures 6A,C). The colorimetric test kit for LDH release again validated the findings obtained from the PI staining (Figure 6D). In addition, we knocked down FTO using a FTO shRNA lentiviral particles. The silencing efficiency and inhibited FTO activity as revealed by elevated global m^6^A level were confirmed (Supplementary Figures S6A,B). We observed that FTO shRNA phenocopied the effects of FTO inhibitor in terms of elevating PI-positive cell death, LDH release, and reducing cell surface area (Figures 6A–D). Taken together, these results indicate that FTO plays a protective role in SUN-induced hiPSC-CM injury.

To further explore the role of FTO in SUN-induced cardiotoxicity, we employed a lentivirus construct to overexpress FTO. After verifying the FTO mRNA and global m^6^A level, we transfected hiPSC-CMs with the FTO lentivirus construct or empty vector. The results showed that FTO overexpression by lentivirus construct successfully induced a ~2-fold upregulation of FTO expression and a ~40% downregulation of global m^6^A level (Supplementary Figures S5A,B). Furthermore, FTO overexpression significantly reduced LDH release and improved cell viability in SUN-challenged hiPSC-CMs (Supplementary Figures S5C,D).

To justify that SUN-altered m^6^A effects are cardiomyocytes specific or not, we treated CMECs and CFs with 60 nM or 10 μM SUN for 18 h, respectively, referring to the concentration and duration reported in previous studies (23, 24). We then measured the global m^6^A level of mRNA and the transcripts levels of FTO mRNA. Interestingly enough, both the CMECs and CFs failed to show significant changes in these two parameters in response to SUN (Supplementary Figures S7A,B,D,E). Furthermore, cotreatment of FTO inhibitor FB23-2 did not alter the reduced cell viability in response to SUN as revealed by CCK-8 assay (Supplementary Figures S7C,F). These results might indicate that the reported effects are cardiomyocytes specific.

## Discussion

In this study, we performed a genome-wide analysis of m^6^A mRNA methylation by MeRIP-seq and RNA-seq in a human stem cell-derived cardiomyocyte model of TKI-induced cardiotoxicity. Our major findings include the following: (a) the global m^6^A level was upregulated in SUN-injured hiPSC-CMs; (b) downregulated FTO as well as upregulated METTL14 and WTAP might account for the increased global m^6^A level in SUN-injured hiPSC-CMs; (c) m^6^A modification was associated with the occurrence and course of TKI-induced cardiotoxicity to some extent; and (d) protected against SUN-induced hiPSC-CM injury. Nevertheless, the specific mechanism of m^6^A methylation in TKI-induced cardiotoxicity remains to be further studied in the future.

Recent studies have shown that m^6^A is involved in the occurrence and development of cancers and cardiac dysfunction. RNA methyltransferases, demethylases, and m^6^A-binding proteins are frequently altered in human cancer tissues from various organ sources, influencing cancer transcription and oncoprotein expression, cancer cell proliferation, survival, tumor initiation, progression, and metastasis (25, 26). However, there is no consensus on whether altered m^6^A is oncogenic or tumor suppressive. In comparison, studies have reported that m^6^A methylation and FTO have decreased expressions in various pathologic conditions including heart failure and endotoxemia- and hypoxia-/reoxygenation-induced cardiac cell injuries (7, 14, 15). In accordance with our present finding, most studies suggest that elevated m^6^A level that result from elevated methylases or reduced demethylases is a deleterious factor for the onset and progression of various cardiovascular diseases (27).

Recently, m^6^A methylation was revealed to play important roles in various cardiovascular diseases, but its role in TKI-induced cardiotoxicity has rarely been studied. In this study, we found that the cell viability of hiPCS-CMs treated with SUN decreased, whereas the release of LDH increased, which suggested that SUN had a damaging effect on hiPSC-CMs. IF staining showed that the m^6^A levels of the SUN group were elevated and the m^6^A dot blot also verified this elevation. Moreover, we also found that the global m^6^A methylation level in SUN-treated hiPSC-CMs was positively correlated with LDH release. Therefore, we employed MeRIP-seq and RNA-seq to study the transcriptome and methylome in SUN-treated hiPSC-CMs. Through further joint analysis of MeRIP-seq and RNA-seq data, we found significant differences between the m^6^A methylation and mRNA expression levels of 244 genes. We surmised that these potential genes, through m^6^A methylation, are potentially involved in the occurrence and development of TKI-induced cardiotoxicity.

We further conducted the GO and the KEGG analyses of differentially expressed m^6^A methylated genes. Biological processes and pathways indicated apoptotic signaling pathways that were repetitiously enriched by upregulated DEGs. This enrichment pattern coincides with previous SUN cardiotoxicity studies. For instance, a previous study in isolated cardiomyocytes and mice scrutinized the potential mechanisms of SUN-associated cardiac effects. This study concluded that mitochondrial injury and cardiomyocyte apoptosis accounted for SUN-associated cardiotoxicity (28). In a more recent report, apoptotic cell death resulting from mitochondrial damage with reactive oxygen species (ROS) accumulation was shown to be the important contributing mechanism of cardiotoxicity associated with SUN (29). Conversely, thioester and acetyl-CoA metabolic pathways were significantly enriched by downregulated DEGs. Thioesters play a prominent role in metabolism. The central metabolite acetyl-CoA is a thioester that is produced mainly by oxidative decarboxylation of pyruvate or by fatty acid degradation. It is, thus, possible that SUN-induced cardiotoxicity can be ascribed to interrupted mitochondrial energy production. Interestingly, SUN was previously reported to induce loss of mitochondrial membrane potential and energy rundown in cardiomyocytes (30). Thus, this study further supports the notion that apoptosis induction and energy reduction are the crucial mechanisms of SUN-associated cardiotoxicity.

Fat mass and obesity-associated protein is the first demethylase discovered to be involved in m^6^A modification. Recent high-quality studies have confirmed that FTO plays fundamental roles in many cardiac physiological and pathological processes. Increased m^6^A in RNA was associated with decreased FTO mRNA and protein expression in human and mouse failing hearts. Moreover, adeno-associated virus 9 (AAV9)-mediated myocardial FTO overexpression restores cardiac function in mouse models of myocardial infarction, whereas cardiomyocyte-restricted knockout of FTO mice deteriorates cardiac function (27). Further mechanistic studies revealed that FTO overexpression selectively demethylates cardiac contractile transcripts, thus blocking their degradation and improving their stability and expression under ischemia, which eventually contributed to reduced fibrosis and enhanced angiogenesis (7). Another study found that FTO alleviated cardiac dysfunction by regulating glucose uptake and glycolysis in mice with pressure overload-induced heart failure, the effects of which were associated with demethylation of the glycolysis-related gene *Pgam2* (31). In addition, a similar study reported that FTO cardiomyocyte-specific knockout worsened cardiac dysfunction through transcription-independent mechanisms of translation regulation (32). All these reports support a cardioprotective role of FTO in different cardiac pathologies. In this study, we detected the major methylases and demethylases in SUN-treated hiPSC-CMs and found that FTO was significantly downregulated. To verify the role of FTO in SUN-induced cardiotoxicity, we treated hiPSC-CMs with FB23-2, a potent and selective FTO inhibitor and demonstrated that FB23-2 can aggravate the cell injury elicited by SUN, cause damage to the sarcomeres of cardiomyocytes, and deteriorate cell atrophy. Together with previous findings, this study might add a conceptual framework for targeting FTO as a therapeutic for various cardiovascular diseases. Notably, this study also found that the expression levels of METTL14 and WTAP increased in SUN-treated hiPSC-CMs and their functional roles remain to be further clarified.

This study failed to reveal the downstream regulatory mechanisms by which SUN-stimulated m^6^A upregulation regulates the mRNA expression of related genes, which warrants further investigations. Nonetheless, we have depicted the m^6^A modification landscape of SUN-treated hiPSC-CMs with transcriptome-wide unbiased epitranscriptomics and revealed a potential role of m^6^A and m^6^A eraser FTO in SUN-induced cardiotoxicity, which would lay a solid foundation for further detailed mechanistic studies.

## Conclusion

This study provides the first overview of the m^6^A methylation map in SUN-injured hiPSC-CMs to decipher the RNA post-transcriptional epigenetic mechanisms of TKI-induced cardiotoxicity. Through MeRIP-seq, we found that the m^6^A methylation level in 2,614 mRNAs changed significantly. Combined analysis of the m^6^A peak and mRNA expression showed that 244 mRNAs were significantly changed after SUN treatment. These genes with varying levels of m^6^A modification may play an important role in the process of TKI-induced cardiotoxicity. In addition, we found that inhibiting FTO can aggravate the myocardial toxicity caused by SUN, which suggests a novel therapeutic target for TKI-induced cardiotoxicity.

## Data Availability Statement

The data presented in the study are deposited in the NCBI Gene Expression Omnibus (GEO) database repository, accession number GSE192913.

## Author Contributions

FC and DH conceived and designed the experiments, provided financial support, co-wrote the article, and edited the manuscript. YM and XL performed the experiments. YM, XL, YB, TW, CC, and YW analyzed the data. All authors contributed to the article and approved the submitted version.

## Funding

This study was supported by the National Natural Science Foundation of China (82100372, 81820108019, and 91939303), the Innovative Project of Chinese PLA General Hospital (CX19028), the Key Health Care Projects of National Health Commission (2020ZD05), National Postdoctoral Program for Innovative Talents (BX20200154), Beijing Nova Program (Z211100002121048), and the Basic Research Reinforcement Project (2021-JCJQ-JJ-1079).

## Conflict of Interest

The authors declare that the research was conducted in the absence of any commercial or financial relationships that could be construed as a potential conflict of interest.

## Publisher's Note

All claims expressed in this article are solely those of the authors and do not necessarily represent those of their affiliated organizations, or those of the publisher, the editors and the reviewers. Any product that may be evaluated in this article, or claim that may be made by its manufacturer, is not guaranteed or endorsed by the publisher.

## Abbreviations

METTL14: methyltransferase-like 14
WTAP: Wilms&#8217
tumor 1-associating protein
ALKBH5: Alk B homologue 5
YTHDF, CYT homology domain family, HNRNPC: heterogeneous nuclear ribonucleo protein C
HNRNPA2B1: heterogeneous nuclear ribonucleoprotein A2B1
EIF3A: eukaryotic initiation factor 3A
RPMI: Roswell Park Memorial Institute
IWR-1: Wnt/β-catenin inhibitor
FB23-2: FTO Demethylase inhibitor
RIPA: Radio-Immunoprecipitation Assay
PVDF: poly vinyli dene fluoride
TBST: Tris-Buffered Saline and Tween
ECL: enhanced chemiluminescence
GAPDH: glyceraldehyde-3- phosphate dehydrogenase
DTT: DL-Dithiothreitol
MIT: Massachusetts Institute of Technology
AAV9: adeno-associated virus 9.
