# Supplementary material for: Alteration of N6-Methyladenosine mRNA Methylation in a Human Stem Cell-Derived Cardiomyocyte Model of Tyrosine Kinase Inhibitor-Induced Cardiotoxicity
Source: Front Cardiovasc Med. 2022 Mar 23;9:849175. doi: 10.3389/fcvm.2022.849175 (PMC8985653; doi:10.3389/fcvm.2022.849175)
Supplement: Supplementary file 1 [file Data_Sheet_1.pdf]

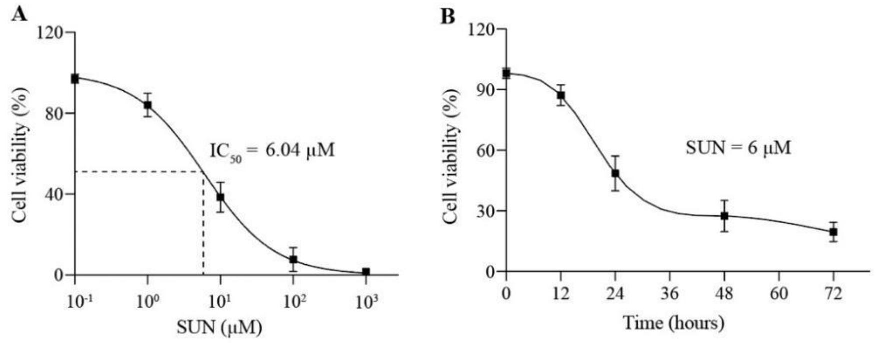

Figure S1. The IC_{50} and time kinetics study of SUN-treated hiPSC-CMs. (A) Cell viability was was assessed by CCK-8 assay after 24h treatment of cells with different concentrations of SUN. (B) SUN (6 μmol/L) hiPSC-CMs were treated with SUN (6 μmol/L) for 6h, 12h, 24h, 48h and 72h, then the cell viability was detected.

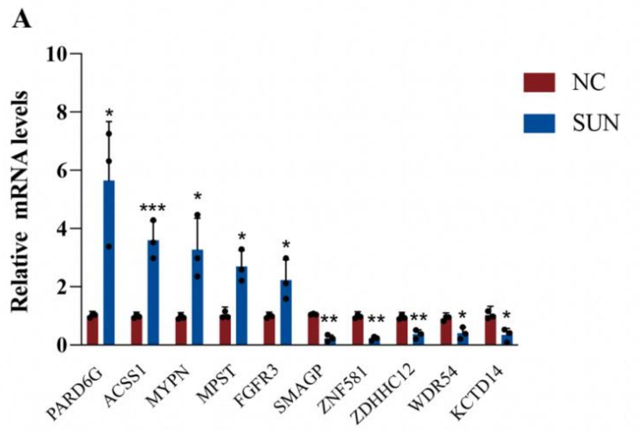

Figure S2. Validation of the top5 up-regulated and top5 down-regulated genes among the 261 intersection genes by qRT-PCR assay. "*" indicates $p < 0.05$, "**" indicates $p < 0.01$, and "***" indicates $p < 0.001$.

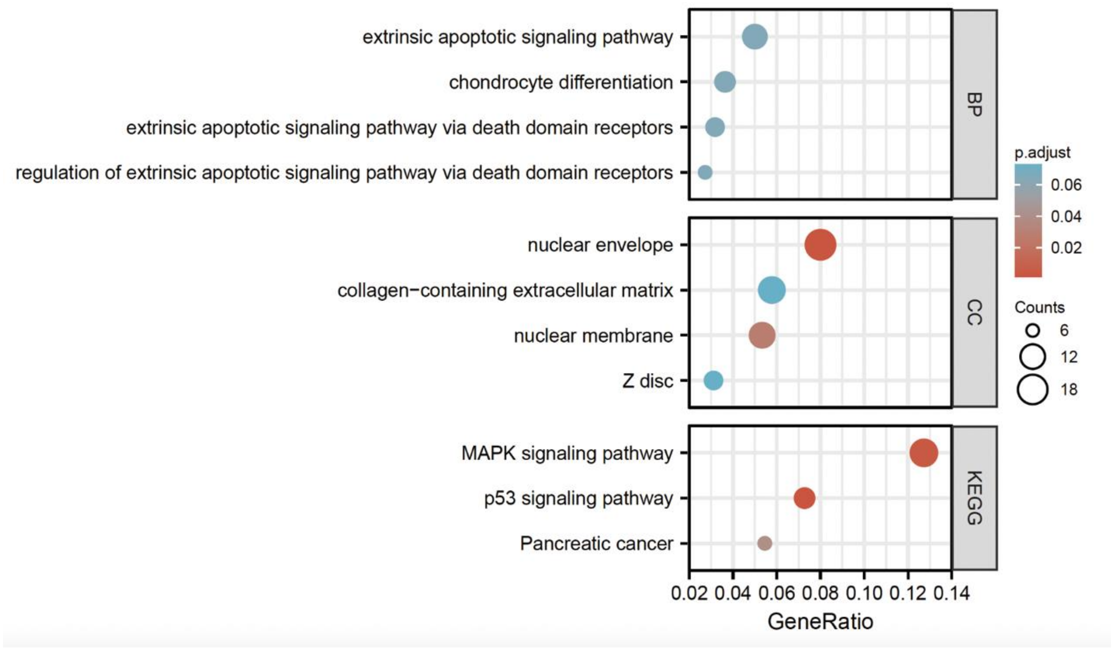

Figure S3. (A) Enrichment analysis of 244 mRNAs with m6A peak changes and expression changes.

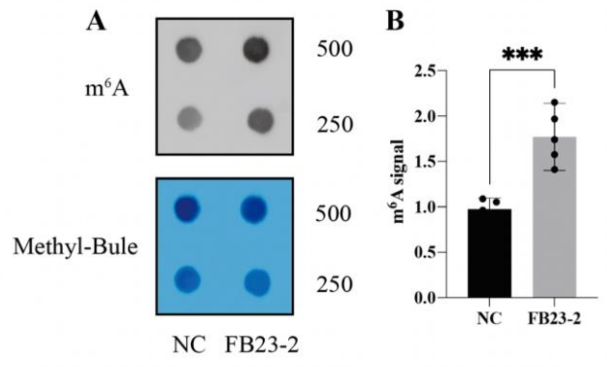

Figure S4. The effect of FB23-2 on the global m6A level. (A) and (B) hiPSC-CMs were treated with 20μM FB23-2 for 24h, and the m6A signal was detected by dot blot. "***" indicates p < 0.001."NC" indicates negative control.

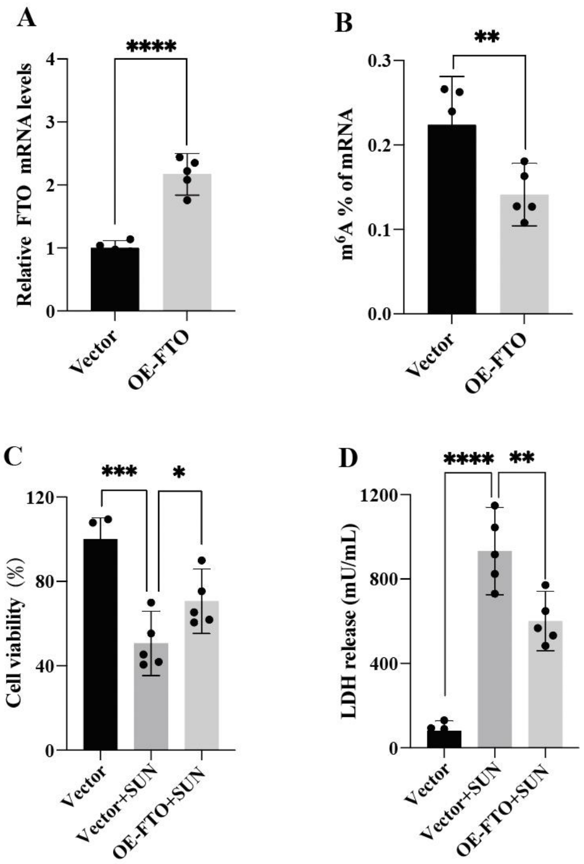

Figure S5. FTO overexpression mitigated SUN-induced cardiotoxicity. (A) qRT-PCR based detection of FTO mRNA levels in FTO overexpressed (OE) hiPSC-CMs and vector groups. (B) Measurement of LDH release level. n=5; "**" indicates P<0.01, "****" indicates P<0.0001. "OE-FTO" stands for FTO overexpressed.

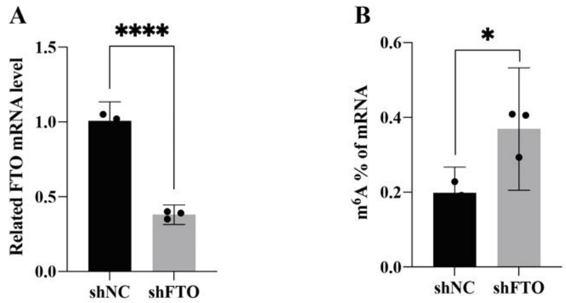

Figure S6. Confirmation of silencing efficiency and inhibited FTO activity as revealed by global m6A level. (A) The histogram shows the expression level of FTO gene in shNC and shFTO groups. (B) "****" indicate p < 0.0001.

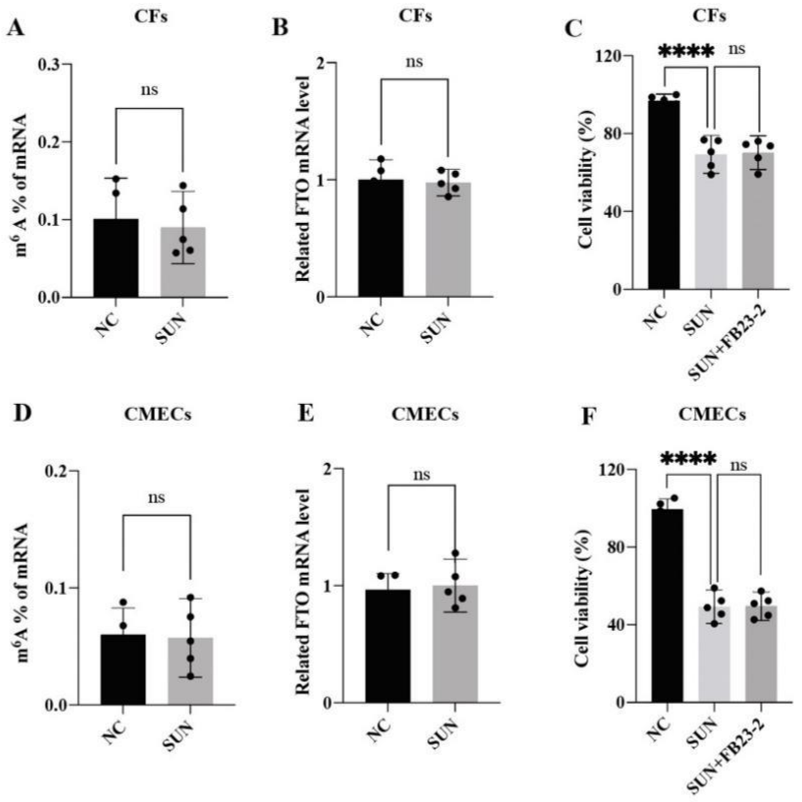

Figure S7. Other cardiac cell types in response to SUN and FTO inhibitor treatment. (A) CMECs and and (B) CFs were treated with 60 nM or 10μM SUN for 18h, respectively, then the global m6A level of mRNA was detected using m6A RNA Methylation Assay Kit (Colorimetric). (C) and (D) The expression of FTO mRNA were detected by qRT-PCR in CMECs and CFs. (E) and (F) Following co-treatment of FTO inhibitor FB23-2 (20μM for 18h) in SUN-treated CMECs and CFs, cell viability was detected by CCK-8 assay. “ns” stands for non-significant. “****” denotes $P < 0.001$.

Supplementary table 1: *The Assay ID for TaqMan® Gene Expression Assays.*

Gene name	Assay ID
GAPDH	Hs02786624_g1
FTO	Hs01057139_g1
ALKBH5	Hs00539502_m1
METTL3	Hs00219820_m1

METTL14	Hs00383340_m1
WTAP	Hs04987070_m1
PARD6G	Hs00261284_m1
ACSS1	Hs00544656_g1
MYPN	Hs00261515_m1
MPST	Hs05579360_s1
FGFR3	Hs00179829_m1
SMAGP	Hs01059487_m1
ZNF581	Hs00255569_s1
ZDHHC12	Hs00982596_g1
WDR54	Hs00259557_m1
KCTD14	Hs01928822_s1

Supplementary table 2:
<u>The list of 244 differentially expressed genes with significant differential m6A peaks.xls</u>

Ensembl_gene_id	Log2FC	External gene name	Fold enrichment	Label
ENSG00000005448	-4.6257083	WDR54	4.4	Hyper-Methylated-down
ENSG00000011566	-1.5501019	MAP4K3	2.45	Hyper-Methylated-down
ENSG00000047849	-1.0305736	MAP4	11	Hyper-Methylated-down
ENSG00000065559	-2.2161057	MAP2K4	39.5	Hyper-Methylated-down
ENSG00000080345	-1.2953207	RIF1	5.85	Hyper-Methylated-down
ENSG00000091986	-2.1288842	CCDC80	11.4	Hyper-Methylated-down
ENSG00000100815	-1.5851595	TRIP11	5.11	Hyper-Methylated-down
ENSG00000101868	-1.1732329	POLA1	4.43	Hyper-Methylated-down
ENSG00000102595	-1.3850626	UGGT2	5.01	Hyper-Methylated-down
ENSG00000105341	-2.2341494	DMAC2	2.71	Hyper-Methylated-down
ENSG00000106077	-4.4219339	ABHD11	17.6	Hyper-Methylated-down
ENSG00000109381	-1.3389281	ELF2	11.5	Hyper-Methylated-down
ENSG00000109390	-2.0954932	NDUFC1	18.3	Hyper-Methylated-down
ENSG00000119408	-1.3739624	NEK6	2.48	Hyper-Methylated-down
ENSG00000124588	-3.9328501	NQO2	5.81	Hyper-Methylated-down
ENSG00000130635	-1.7125232	COL5A1	24.3	Hyper-Methylated-down
ENSG00000131127	-2.1634284	ZNF141	33	Hyper-Methylated-down
ENSG00000136010	-1.3027975	ALDH1L2	25.7	Hyper-Methylated-down

ENSG00000147905	-1.9688681	ZCCHC7	5.34	Hyper-Methylated-down
ENSG00000149257	-1.2406048	SERPINH1	2.02	Hyper-Methylated-down
ENSG00000151304	-2.3586693	SRFBP1	42.3	Hyper-Methylated-down
ENSG00000151353	-3.3858377	TMEM18	5.38	Hyper-Methylated-down
ENSG00000151364	-4.5434503	KCTD14	23	Hyper-Methylated-down
ENSG00000152253	-2.053054	SPC25	3.67	Hyper-Methylated-down
ENSG00000157764	-1.0700584	BRAF	12.6	Hyper-Methylated-down
ENSG00000160446	-5.1092492	ZDHHC12	51.3	Hyper-Methylated-down
ENSG00000163946	-1.6231714	FAM208A	16.8	Hyper-Methylated-down
ENSG00000165671	-1.1363958	NSD1	29.5	Hyper-Methylated-down
ENSG00000166579	-1.3312694	NDEL1	18.1	Hyper-Methylated-down
ENSG00000182670	-1.2802525	TTC3	19.6	Hyper-Methylated-down
ENSG00000182704	-2.27483	TSKU	24.1	Hyper-Methylated-down
ENSG00000185104	-1.7028787	FAF1	3.51	Hyper-Methylated-down
ENSG00000185669	-4.4099821	SNAI3	114	Hyper-Methylated-down
ENSG00000188994	-1.2872973	ZNF292	21.6	Hyper-Methylated-down
ENSG00000198015	-1.3003727	MRPL42	8.38	Hyper-Methylated-down
ENSG00000205581	-1.1114775	HMGN1	1.73	Hyper-Methylated-down
ENSG00000278463	-2.8236284	HIST1H2AB	4.08	Hyper-Methylated-down
ENSG00000003400	3.65989034	CASP10	8.38	Hyper-Methylated-up
ENSG00000012171	2.65185596	SEMA3B	9.69	Hyper-Methylated-up
ENSG00000026036	6.76983802	RTEL1-TNFRSF6B	5.5	Hyper-Methylated-up
ENSG00000049130	2.46364544	KITLG	12.6	Hyper-Methylated-up
ENSG00000063587	4.14527209	ZNF275	36.7	Hyper-Methylated-up
ENSG00000064601	1.0018414	CTSA	44.5	Hyper-Methylated-up
ENSG00000066422	1.18302563	ZBTB11	7.07	Hyper-Methylated-up
ENSG00000068078	5.4466842	FGFR3	13.7	Hyper-Methylated-up
ENSG00000072786	1.15389637	STK10	1.9	Hyper-Methylated-up
ENSG00000074527	1.40186961	NTN4	5.79	Hyper-Methylated-up
ENSG00000079335	2.77011761	CDC14A	3.81	Hyper-Methylated-up
ENSG00000089486	1.48147325	CDIP1	9.35	Hyper-Methylated-up
ENSG00000090054	1.27264334	SPTLC1	2.53	Hyper-Methylated-up
ENSG00000092054	3.54154514	MYH7	71.8	Hyper-Methylated-up
ENSG00000103066	2.87298117	PLA2G15	31.8	Hyper-Methylated-up
ENSG00000103404	1.89733917	USP31	4.4	Hyper-Methylated-up
ENSG00000105939	1.07292005	ZC3HAV1	4.54	Hyper-Methylated-up
ENSG00000106009	1.75404665	BRAT1	24.1	Hyper-Methylated-up
ENSG00000106546	2.54550295	AHR	17.6	Hyper-Methylated-up
ENSG00000114270	1.67477547	COL7A1	8.9	Hyper-Methylated-up
ENSG00000121690	2.88583672	DEPDC7	9.19	Hyper-Methylated-up
ENSG00000124067	1.84138368	SLC12A4	15	Hyper-Methylated-up
ENSG00000124226	1.87742592	RNF114	2.25	Hyper-Methylated-up
ENSG00000125538	2.28522413	IL1B	12.4	Hyper-Methylated-up

ENSG00000126368	5.11608208	NR1D1	7.51	Hyper-Methylated-up
ENSG00000127124	2.76693461	HIVEP3	10.8	Hyper-Methylated-up
ENSG00000128510	1.59708108	CPA4	4.11	Hyper-Methylated-up
ENSG00000133731	3.03963115	IMPA1	7.85	Hyper-Methylated-up
ENSG00000135823	2.80532897	STX6	6.9	Hyper-Methylated-up
ENSG00000138347	6.35776403	MYPN	38.4	Hyper-Methylated-up
ENSG00000143878	1.23925973	RHOB	34	Hyper-Methylated-up
ENSG00000151131	2.68268734	C12orf45	25.1	Hyper-Methylated-up
ENSG00000154736	3.5366944	ADAMTS5	17.1	Hyper-Methylated-up
ENSG00000154930	6.7269564	ACSS1	4.84	Hyper-Methylated-up
ENSG00000155330	2.43886303	C16orf87	9.73	Hyper-Methylated-up
ENSG00000156471	1.94154833	PTDSS1	6.28	Hyper-Methylated-up
ENSG00000158615	1.64396756	PPP1R15B	2.92	Hyper-Methylated-up
ENSG00000159873	2.45686488	CCDC117	5.67	Hyper-Methylated-up
ENSG00000163788	1.69181729	SNRK	11	Hyper-Methylated-up
ENSG00000167196	1.36236833	FBXO22	16.5	Hyper-Methylated-up
ENSG00000167378	2.53449342	IRGQ	2.35	Hyper-Methylated-up
ENSG00000168685	1.43177031	IL7R	19.4	Hyper-Methylated-up
ENSG00000168724	1.21371075	DNAJC21	22	Hyper-Methylated-up
ENSG00000170385	1.42600666	SLC30A1	9.26	Hyper-Methylated-up
ENSG00000170471	1.32589185	RALGAPB	63.6	Hyper-Methylated-up
ENSG00000171617	1.40800565	ENC1	14.7	Hyper-Methylated-up
ENSG00000172667	1.79056987	ZMAT3	47.2	Hyper-Methylated-up
ENSG00000172890	1.52720329	NADSYN1	10.5	Hyper-Methylated-up
ENSG00000173276	1.77160505	ZBTB21	11	Hyper-Methylated-up
ENSG00000178184	8.48945727	PARD6G	34.6	Hyper-Methylated-up
ENSG00000185567	1.38628074	AHNAK2	25.1	Hyper-Methylated-up
ENSG00000197044	1.57492084	ZNF441	8.66	Hyper-Methylated-up
ENSG00000197530	2.07236917	MIB2	6.94	Hyper-Methylated-up
ENSG00000197852	2.19951293	FAM212B	22.3	Hyper-Methylated-up
ENSG00000213625	1.46962567	LEPROT	4.08	Hyper-Methylated-up
ENSG00000007202	-1.1573533	KIAA0100	18.1	Hypo-Methylated-down
ENSG00000024526	-2.0389589	DEPDC1	54.1	Hypo-Methylated-down
ENSG00000044459	-2.5399679	CNTLN	13.4	Hypo-Methylated-down
ENSG00000048471	-1.3219721	SNX29	13.9	Hypo-Methylated-down
ENSG00000054118	-1.1953839	THRAP3	3.93	Hypo-Methylated-down
ENSG00000059804	-1.9763386	SLC2A3	2.86	Hypo-Methylated-down
ENSG00000060982	-2.6625894	BCAT1	2.62	Hypo-Methylated-down
ENSG00000065060	-1.947907	UHRF1BP1	14.7	Hypo-Methylated-down
ENSG00000066279	-1.2238438	ASPM	21.3	Hypo-Methylated-down
ENSG00000066455	-1.3524137	GOLGA5	19.4	Hypo-Methylated-down
ENSG00000067141	-2.096662	NEO1	2.62	Hypo-Methylated-down
ENSG00000071794	-1.3778748	HLTF	7.1	Hypo-Methylated-down

ENSG00000077254	-1.2206526	USP33	2.36	Hypo-Methylated-down
ENSG00000078142	-1.2933281	PIK3C3	9.95	Hypo-Methylated-down
ENSG00000087253	-2.4883652	LPCAT2	17.3	Hypo-Methylated-down
ENSG00000090863	-1.4507102	GLG1	6.55	Hypo-Methylated-down
ENSG00000091436	-1.8542565	MAP3K20	9.16	Hypo-Methylated-down
ENSG00000100629	-1.8117539	CEP128	25	Hypo-Methylated-down
ENSG00000100714	-1.0542644	MTHFD1	6.11	Hypo-Methylated-down
ENSG00000101346	-1.7225343	POFUT1	10.3	Hypo-Methylated-down
ENSG00000101972	-1.2676213	STAG2	12.5	Hypo-Methylated-down
ENSG00000104093	-1.0180604	DMXL2	39.3	Hypo-Methylated-down
ENSG00000104447	-1.8072372	TRPS1	30.9	Hypo-Methylated-down
ENSG00000104765	-1.0341913	BNIP3L	6.84	Hypo-Methylated-down
ENSG00000106105	-1.0065481	GARS	13.3	Hypo-Methylated-down
ENSG00000111897	-1.0666965	SERINC1	3.9	Hypo-Methylated-down
ENSG00000115365	-2.6841199	LANCL1	17.3	Hypo-Methylated-down
ENSG00000122966	-1.0815634	CIT	11.5	Hypo-Methylated-down
ENSG00000124920	-1.9691627	MYRF	5.06	Hypo-Methylated-down
ENSG00000128340	-2.0964284	RAC2	2.05	Hypo-Methylated-down
ENSG00000129353	-2.4767125	SLC44A2	7.07	Hypo-Methylated-down
ENSG00000129534	-1.8714419	MIS18BP1	5.03	Hypo-Methylated-down
ENSG00000131747	-1.0593482	TOP2A	6.5	Hypo-Methylated-down
ENSG00000132356	-1.3330242	PRKAA1	5.06	Hypo-Methylated-down
ENSG00000133704	-1.6968352	IPO8	6.37	Hypo-Methylated-down
ENSG00000136451	-1.1722367	VEZF1	17.7	Hypo-Methylated-down
ENSG00000136628	-1.3891827	EPRS	5.59	Hypo-Methylated-down
ENSG00000137770	-1.0347434	CTDSPL2	2.22	Hypo-Methylated-down
ENSG00000137809	-1.6615677	ITGA11	4.89	Hypo-Methylated-down
ENSG00000138246	-1.2750778	DNAJC13	23.6	Hypo-Methylated-down
ENSG00000138604	-2.5659832	GLCE	2.92	Hypo-Methylated-down
ENSG00000140694	-1.4887971	PARN	15	Hypo-Methylated-down
ENSG00000140836	-1.1493114	ZFHX3	24.7	Hypo-Methylated-down
ENSG00000142949	-1.3616485	PTPRF	2.51	Hypo-Methylated-down
ENSG00000143228	-2.7578942	NUF2	5.18	Hypo-Methylated-down
ENSG00000144724	-2.298481	PTPRG	4.06	Hypo-Methylated-down
ENSG00000145439	-2.6478705	CBR4	2.59	Hypo-Methylated-down
ENSG00000146463	-1.0464533	ZMYM4	6.89	Hypo-Methylated-down
ENSG00000152377	-1.0877422	SPOCK1	2.4	Hypo-Methylated-down
ENSG00000152661	-1.0571648	GJA1	2.58	Hypo-Methylated-down
ENSG00000152818	-1.0167551	UTRN	6.02	Hypo-Methylated-down
ENSG00000155827	-1.2567042	RNF20	10.5	Hypo-Methylated-down
ENSG00000156162	-2.0816911	DPY19L4	2.55	Hypo-Methylated-down
ENSG00000159164	-2.4302567	SV2A	11.5	Hypo-Methylated-down
ENSG00000162378	-1.3162567	ZYG11B	5.76	Hypo-Methylated-down

ENSG00000163872	-1.1563585	YEATS2	4.11	Hypo-Methylated-down
ENSG00000164244	-1.3793724	PRRC1	15.4	Hypo-Methylated-down
ENSG00000164692	-1.0820526	COL1A2	9.1	Hypo-Methylated-down
ENSG00000165898	-4.4100308	ISCA2	9.63	Hypo-Methylated-down
ENSG00000166888	-1.4860636	STAT6	1.67	Hypo-Methylated-down
ENSG00000168077	-2.8940134	SCARA3	25.1	Hypo-Methylated-down
ENSG00000168172	-1.1299617	HOOK3	3.2	Hypo-Methylated-down
ENSG00000170545	-6.8543712	SMAGP	16.2	Hypo-Methylated-down
ENSG00000171425	-5.6714274	ZNF581	5.14	Hypo-Methylated-down
ENSG00000171456	-1.3810076	ASXL1	19.9	Hypo-Methylated-down
ENSG00000174501	-1.289471	ANKRD36C	3.4	Hypo-Methylated-down
ENSG00000176225	-1.5651316	RTTN	4.01	Hypo-Methylated-down
ENSG00000183621	-2.9081384	ZNF438	15.7	Hypo-Methylated-down
ENSG00000186468	-1.0228057	RPS23	17.8	Hypo-Methylated-down
ENSG00000187118	-2.2925156	CMC1	10.5	Hypo-Methylated-down
ENSG00000196159	-1.4749	FAT4	45.9	Hypo-Methylated-down
ENSG00000196700	-2.5858625	ZNF512B	3.05	Hypo-Methylated-down
ENSG00000196968	-1.3301375	FUT11	11.5	Hypo-Methylated-down
ENSG00000242498	-1.862935	ARPIN	7.94	Hypo-Methylated-down
ENSG00000013588	1.4933604	GPRC5A	3.06	Hypo-Methylated-up
ENSG00000040531	2.06438858	CTNS	9.01	Hypo-Methylated-up
ENSG00000048392	1.50241519	RRM2B	8.83	Hypo-Methylated-up
ENSG00000054267	1.01117125	ARID4B	4.58	Hypo-Methylated-up
ENSG00000063660	1.39642222	GPC1	19.6	Hypo-Methylated-up
ENSG00000071994	1.73309694	PDCD2	3.77	Hypo-Methylated-up
ENSG00000076351	1.09243602	SLC46A1	3.8	Hypo-Methylated-up
ENSG00000079432	1.23618149	CIC	29.5	Hypo-Methylated-up
ENSG00000090674	1.96481411	MCOLN1	8.82	Hypo-Methylated-up
ENSG00000092969	1.6613536	TGFB2	15.1	Hypo-Methylated-up
ENSG00000100439	4.07608083	ABHD4	3.22	Hypo-Methylated-up
ENSG00000100664	1.03954739	EIF5	5.06	Hypo-Methylated-up
ENSG00000103266	2.34185017	STUB1	2.67	Hypo-Methylated-up
ENSG00000103326	1.79799168	CAPN15	2.03	Hypo-Methylated-up
ENSG00000104728	3.31915411	ARHGEF10	3.52	Hypo-Methylated-up
ENSG00000105339	2.25309206	DENND3	2.73	Hypo-Methylated-up
ENSG00000106366	1.65669548	SERPINE1	7.45	Hypo-Methylated-up
ENSG00000107937	1.44840498	GTPBP4	3.14	Hypo-Methylated-up
ENSG00000109790	1.11397442	KLHL5	13.2	Hypo-Methylated-up
ENSG00000111727	1.48838284	HCFC2	14.6	Hypo-Methylated-up
ENSG00000113739	1.16347923	STC2	2.75	Hypo-Methylated-up
ENSG00000116717	2.94850611	GADD45A	6.62	Hypo-Methylated-up
ENSG00000118503	1.90567697	TNFAIP3	8.98	Hypo-Methylated-up
ENSG00000119801	1.70468832	YPEL5	4.06	Hypo-Methylated-up

ENSG00000120451	1.55851859	SNX19	4.23	Hypo-Methylated-up
ENSG00000120868	2.13682049	APAF1	5.18	Hypo-Methylated-up
ENSG00000120889	1.89236829	TNFRSF10B	9.99	Hypo-Methylated-up
ENSG00000123983	1.32238814	ACSL3	6.63	Hypo-Methylated-up
ENSG00000124201	1.1729959	ZNFX1	32.3	Hypo-Methylated-up
ENSG00000124789	1.09013389	NUP153	4.71	Hypo-Methylated-up
ENSG00000126003	1.66082765	PLAGL2	12.8	Hypo-Methylated-up
ENSG00000128283	1.70754303	CDC42EP1	4.18	Hypo-Methylated-up
ENSG00000128309	5.66623927	MPST	7	Hypo-Methylated-up
ENSG00000128591	1.42877458	FLNC	4.65	Hypo-Methylated-up
ENSG00000130513	3.39271626	GDF15	8.46	Hypo-Methylated-up
ENSG00000130589	1.73711959	HELZ2	5.31	Hypo-Methylated-up
ENSG00000132819	4.43127606	RBM38	15.7	Hypo-Methylated-up
ENSG00000134452	1.53515184	FBXO18	19.4	Hypo-Methylated-up
ENSG00000136732	2.94983189	GYPC	27.4	Hypo-Methylated-up
ENSG00000140396	1.68316133	NCOA2	34.2	Hypo-Methylated-up
ENSG00000141682	2.96972406	PMAIP1	15	Hypo-Methylated-up
ENSG00000142627	3.52850738	EPHA2	5.99	Hypo-Methylated-up
ENSG00000146574	5.26500171	CCZ1B	2.98	Hypo-Methylated-up
ENSG00000148400	1.51864861	NOTCH1	8.46	Hypo-Methylated-up
ENSG00000150347	1.02734066	ARID5B	2.29	Hypo-Methylated-up
ENSG00000151929	1.40723387	BAG3	2.98	Hypo-Methylated-up
ENSG00000152127	1.34012268	MGAT5	2.51	Hypo-Methylated-up
ENSG00000155438	2.17988941	NIFK	18.3	Hypo-Methylated-up
ENSG00000156983	1.89396579	BRPF1	3.27	Hypo-Methylated-up
ENSG00000157350	2.16967491	ST3GAL2	91.6	Hypo-Methylated-up
ENSG00000159082	2.29769342	SYNJ1	11.4	Hypo-Methylated-up
ENSG00000163874	2.19119165	ZC3H12A	7.78	Hypo-Methylated-up
ENSG00000164609	1.5977304	SLU7	6.05	Hypo-Methylated-up
ENSG00000165802	1.82537404	NSMF	1.34	Hypo-Methylated-up
ENSG00000166881	1.38708936	NEMP1	2.28	Hypo-Methylated-up
ENSG00000168610	1.03256744	STAT3	2.94	Hypo-Methylated-up
ENSG00000168994	1.7617621	PXDC1	4.43	Hypo-Methylated-up
ENSG00000171552	1.5258493	BCL2L1	2.93	Hypo-Methylated-up
ENSG00000173391	2.98700277	OLR1	14.7	Hypo-Methylated-up
ENSG00000173482	1.60073672	PTPRM	7.3	Hypo-Methylated-up
ENSG00000173511	3.20560716	VEGFB	8.9	Hypo-Methylated-up
ENSG00000173530	2.11388351	TNFRSF10D	15.2	Hypo-Methylated-up
ENSG00000173786	1.02333919	CNP	1.7	Hypo-Methylated-up
ENSG00000175137	2.76910191	SH3BP5L	4.99	Hypo-Methylated-up
ENSG00000175197	2.34576359	DDIT3	7.33	Hypo-Methylated-up
ENSG00000177169	2.21763194	ULK1	15.6	Hypo-Methylated-up
ENSG00000178927	2.79836832	C17orf62	20.4	Hypo-Methylated-up

ENSG00000181026	3.96667865	AEN	3.2	Hypo-Methylated-up
ENSG00000182831	1.37191973	C16orf72	4.71	Hypo-Methylated-up
ENSG00000184436	4.16828803	THAP7	20.2	Hypo-Methylated-up
ENSG00000185414	4.75546909	MRPL30	9.95	Hypo-Methylated-up
ENSG00000189067	1.87195628	LITAF	2.9	Hypo-Methylated-up
ENSG00000196352	1.39658217	CD55	7.33	Hypo-Methylated-up
ENSG00000198142	3.59492761	SOWAHC	12.2	Hypo-Methylated-up
ENSG00000205531	1.21198666	NAP1L4	4.79	Hypo-Methylated-up
ENSG00000246705	1.90687807	H2AFJ	3.72	Hypo-Methylated-up
ENSG00000259330	2.90841736	INAFM2	32.5	Hypo-Methylated-up
ENSG00000266412	1.00221836	NCOA4	4.72	Hypo-Methylated-up
